# Metformin Impairs Breast Cancer Growth through the Inhibition of PRMT6

**DOI:** 10.1002/advs.202508525

**Published:** 2025-12-01

**Authors:** Yinsheng Wu, Xinlin Xu, Yue Tong, Min Wang, Feng Ge, Min Wu, Yunlong Wang, Gang Chen, Xilan Yu, Shanshan Li

**Affiliations:** ^1^ School of Life Sciences Hubei University Wuhan Hubei 430062 China; ^2^ Key Laboratory of Algal Biology Institute of Hydrobiology Chinese Academy of Sciences Wuhan Hubei 430072 China; ^3^ College of Life Sciences Wuhan University Wuhan Hubei 430072 China; ^4^ Hubei University of Chinese Medicine Wuhan 430065 P. R. China; ^5^ Department of Geriatrics Hubei Provincial Hospital of Traditional Chinese Medicine Affiliated Hospital of Hubei University of Chinese Medicine Wuhan 430061 P. R. China; ^6^ Hubei Shizhen Laboratory Wuhan 430065 P. R. China

**Keywords:** breast cancer, DNA methylation, DNA replication, H3R2me2a, Metformin, PRMT6

## Abstract

Metformin is the world's widely prescribed oral medication for the treatment of type 2 diabetes mellitus, which is also shown to repress tumorigenesis without a complete understanding of its therapeutic targets. Here, it is shown that metformin impairs the growth of breast cancer cells by inhibiting PRMT6, a protein arginine methyltransferase primarily responsible for asymmetric dimethylation of histone H3 arginine 2 (H3R2me2a). Mechanistically, metformin directly binds PRMT6 and inhibits its activity to methylate H3R2, leading to *PRMT6* transcriptional repression and further reduces H3R2me2a. By decreasing PRMT6‐catalyzed H3R2me2a, metformin enhances the chromatin association of UHRF1, an accessory factor of DNMT1 to promote DNA methylation and repress the transcription of DNA replication‐associated genes, resulting in retarded DNA replication and cell cycle arrest. Metformin and a DNA replication inhibitor synergistically inhibit tumor growth. Furthermore, genetic disruption of the interaction between metformin and PRMT6 attenuates the inhibitory effect of metformin on breast cancer growth. Together, this work identifies a previously unrecognized mechanism for metformin to inhibit breast cancer growth.

## Graphical abstract

Metformin has a biological activity against breast cancer. However, it is largely unknown about its precise therapeutic targets. Here, histone arginine methyltransferase PRMT6 is identified as a new anti‐cancer target for metformin. Metformin directly binds PRMT6 and inhibits its ability to catalyze histone H3R2 asymmetric dimethylation (H3R2me2a), which then enhances DNMT1‐catalyzed DNA methylation (5mC), resulting in DNA replication defects and cell cycle arrest.

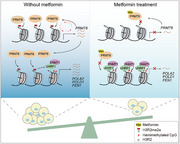

## 1 Introduction

Breast cancer is the most commonly diagnosed cancer worldwide with an estimated 2.3 million new cases annual (11.7%), and represents a significant public health concern.^[^ ^1^ ^]^ The aetiology behind breast cancer involves interactions between environmental, lifestyle, and genetic factors that collectively determine cancer risk.^[^ ^2^ ^]^ The growth of breast cancer is intimately connected to glucose metabolism, and comparative cohort studies and case‐control studies indicate that type 2 diabetes increases the relative risk of breast cancer by 10–20%.^[^ ^3^, ^4^ ^]^ In addition, diabetes and its complications adversely affect cancer therapy and thus influence the outcome of patients with breast cancer.^[^ ^4^ ^]^ In these patients, the use of anti‐diabetic drug metformin has been reported to decrease cancer incidence, and improve the prognosis of breast cancer; thus metformin is considered as a potential adjuvant for the management of breast cancer.^[^ ^5^ ^]^ The biguanide metformin is the world's widely prescribed oral medication for the treatment of type 2 diabetes mellitus at a low cost. Metformin reduces insulin resistance and diabetes‐related morbidity and mortality.^[^ ^6^ ^]^ Metformin has biological activity against all estrogen receptor (ER)‐positive and ER‐negative breast cancer cells.^[^ ^7^ ^]^ Compared with healthy breast tissue, breast cancer tissue has a greater exposure to metformin, suggesting a direct anti‐neoplastic effect of metformin on breast cancer.^[^ ^8^ ^]^ However, the precise therapeutic and biological targets of metformin are largely unknown.^[^ ^9^ ^]^

The extensively studied effector of metformin is AMP‐activated protein kinase (AMPK), a central sensor of cell energetic status.^[^ ^10^, ^11^ ^]^ Metformin inhibits complex I of the electron transport chain, leading to decreased adenosine triphosphate (ATP) synthesis and an elevated cellular AMP/ATP ratio, which activates AMPK. A recent study by Ma and colleagues revealed that metformin binds presenilin enhancer 2 (PEN2), a subunit of γ‐secretase to activate AMPK.^[^ ^12^ ^]^ Activated AMPK then inhibits the mammalian target of rapamycin (mTOR) to reduce overall protein synthesis, cell cycle progression, cell proliferation, and angiogenesis.^[^ ^13^, ^14^ ^]^ Metformin has also been shown to inhibit mTOR, regulate glucose metabolism, and impair cell growth independent of AMPK,^[^ ^15^, ^16^, ^17^ ^]^ suggesting the existence of AMPK‐independent targets for metformin. Moreover, Palma et al. reported that AMPK activation is not responsible for the anti‐tumor activity of metformin in breast cancer cells.^[^ ^18^ ^]^ The differential effect of metformin among tissue types indicates the existence of an AMPK‐independent mechanism of action for this anti‐diabetic drug.^[^ ^11^ ^]^

Epigenetic modifications, such as DNA methylation and histone post‐translational modifications, play critical roles in cancer cell proliferation, apoptosis, and/or motility by regulating gene expression.^[^ ^14^, ^19^ ^]^ Aberrant DNA methylation patterns, including global hypomethylation and regional hypermethylation, are associated with cancer and implicated in oncogenic events.^[^ ^20^ ^]^ Metformin has been reported to induce DNA hypermethylation by interfering with cell metabolism.^[^ ^21^ ^]^ Metformin decreases the synthesis of S‐adenosylhomocysteine (SAH), a strong feedback inhibitor of DNA methyltransferases, while promotes the accumulation of S‐adenosylmethionine (SAM), a universal methyl donor.^[^ ^21^ ^]^ In addition, metformin has been reported to affect histone H3K27me3 and H3K4me3 in prostate organoids and *Caenorhabditis elegans*, respectively.^[^ ^22^, ^23^ ^]^ Metformin also induces histone acetylation via regulation of intracellular acetyl‐CoA levels.^[^ ^24^ ^]^ However, little is known about how metformin regulates specific types of epigenetic modifications and whether this regulation contributes to its anti‐tumor activity in breast cancer.

Protein arginine methyltransferase 6 (PRMT6) is a type 1 PRMT that catalyzes asymmetric dimethylation of histone H3R2 (H3R2me2a).^[^ ^25^ ^]^ It is frequently overexpressed in a variety of cancer cells, including breast cancer, prostate cancer, bladder tumors, gastric cancer, and lung cancer cells, and its expression is implicated in tumor malignancy.^[^ ^26^, ^27^, ^28^, ^29^, ^30^ ^]^ In this work, we characterize PRMT6 as a new target for metformin to impede breast cancer growth. Metformin directly binds PRMT6 and inhibits its ability to catalyze H3R2me2a. We also identify a feed‐forward loop by which PRMT6 amplifies its own transcription and promotes H3R2me2a. By primarily inhibiting this feed‐forward loop, metformin increases global DNA methylation, represses the transcription of DNA replication‐related genes, and impairs tumorigenesis. Ablation of the PRMT6‐metformin interaction ameliorated the detrimental effect of metformin on cell proliferation and tumorigenesis. Our work thus uncovers a new mechanism by which metformin inhibits tumorigenesis.

## 2 Results

### 2.1 Metformin Impairs the Proliferation of Breast Cancer Cells Independent on AMPK

We compared the effect of metformin on the proliferation of different breast cancer cell lines including triple negative (MDA‐MB‐436, MDA‐MB‐468, and MDA‐MB‐231), estrogen sensitive (T47D), and estrogen receptor alpha (ERα)‐positive MCF7 cell lines. Metformin significantly inhibited the growth of these cells with the effect being greater in some cell lines than others (Figure S1A,B, Supporting Information). The inhibitory effect of metformin was also observed using a breast cancer mouse xenograft model and mouse mammary tumor virus (MMTV) transgenic breast tumor model (Figure S1C,D, Supporting Information).

**Figure 1:**
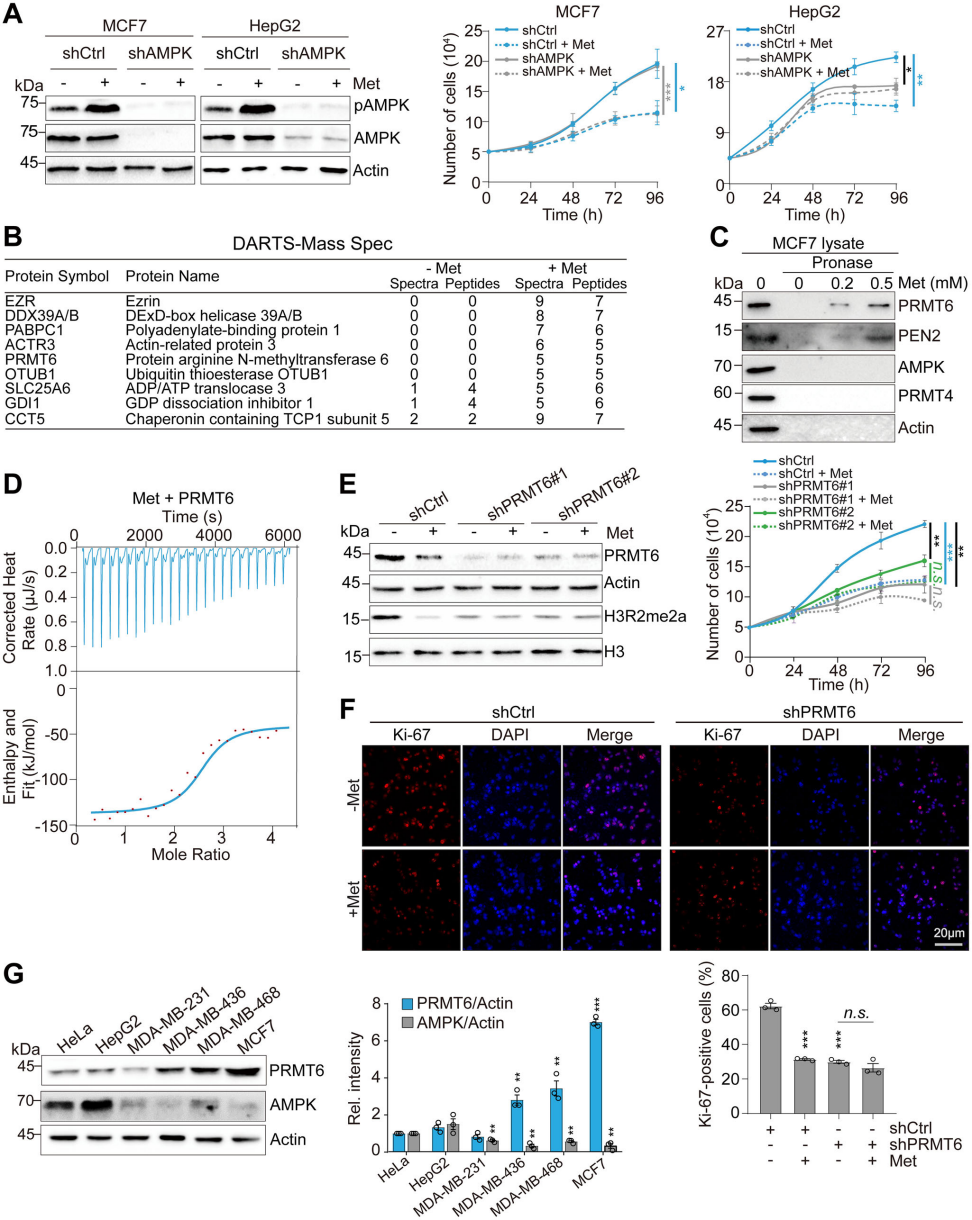
Metformin inhibits breast cancer cell growth primarily by binding to PRMT6. A) Depletion of AMPK attenuated the inhibitory effect of metformin on the growth of HepG2 but not MCF7 cells. Control (shCtrl) and *AMPK*‐knockdown (shAMPK) MCF7 and HepG2 cells were grown in MEM medium supplemented with or without 1 mM metformin (Met). Left panel, immunoblot analysis of *AMPK*‐knockdown efficiency and AMPK activation (pAMPK) by metformin. AMPK activation was assessed using the antibody against AMPK phosphorylated at Thr172 (pAMPK). B) Mass spectrometry analysis of pronase‐digested proteins in control (−Met) and metformin‐treated (+Met) MCF7 lysates. C) Immunoblot analysis of pronase‐digested proteins in MCF7 cell lysates treated with 0–0.5 mm metformin. D) ITC analysis of the interaction between metformin and purified recombinant PRMT6. E,F) Growth curves and Ki‐67 staining of control (shCtrl) and *PRMT6*‐knockdown (shPRMT6#1, shPRMT6#2) MCF7 cells in MEM medium supplemented with or without 1 mM metformin. G) Immunoblots of PRMT6 and AMPK in the indicated cell lines. The relative intensity of PRMT6/Actin and AMPK/Actin was set at 1 in HeLa cells. For (A,E–G), data represent means ± SEM; *n* = 3 biological independent experiments; two‐tailed *t*‐tests (G) and two‐tailed paired *t*‐tests (A,E) were used for statistical analysis. * *P *< 0.05, ** *P *< 0.01, *** *P *< 0.001, *n.s*., no significance. For (C,D), shown are the typical examples of three biological independent experiments.

We then investigated whether metformin inhibits breast cancer cell growth via AMPK. Although metformin activated AMPK in MCF7 cells as indicated by increased AMPK phosphorylation (pAMPK) (Figure S1E, Supporting Information), knockdown of *AMPK* using AMPKα1/α2 short hairpin RNAs (shRNA) in MCF7 and MDA‐MB‐468 cells did not attenuate the inhibitory effect of metformin on cell proliferation (**Figure** **1**A; Figure S1E, Supporting Information). As a control, knockdown of *AMPK* abrogated the detrimental effect of metformin on HepG2 proliferation (Figure 1A), consistent with the reported results^[^ ^31^ ^]^, suggesting that the role of AMPK in metformin‐induced growth impairment depends on the cellular context. Metformin has recently been reported to bind PEN2, a subunit of γ‐secretase to activate AMPK.^[^ ^12^ ^]^ However, knockdown of *PEN2* negligibly affected the inhibitory effect of metformin on MCF7 cell growth (Figure S1F, Supporting Information), further confirming that AMPK is dispensable for metformin to repress MCF7 cell growth. Moreover, AMPK expression in MCF7 was notably lower than that in HepG2 cells (Figure S1G, Supporting Information), suggesting that the differential expression of AMPK in HepG2 and MCF7 cells correlates with their different reliance on AMPK upon metformin treatment.

### 2.2 PRMT6 is Indispensable for Metformin to Inhibit the Proliferation of Breast Cancer Cells

We then aimed to identify the target protein(s) by which metformin represses breast cancer growth. We employed the drug affinity responsive target stability (DARTS) technique combined with mass spectrometry, which is based on the principle that the binding of a small molecule to a target protein can make the protein resistant to protease digestion.^[^ ^32^, ^33^ ^]^ Mass spectrometry identified 9 proteins that were potentially bound and protected by metformin (Figure 1B). Among these candidates, PRMT6 is a histone methyltransferase that catalyzes asymmetric dimethylation of H3R2 (H3R2me2a).^[^ ^25^ ^]^ Moreover, PRMT6 is frequently overexpressed in a variety of cancer cells and its expression is implicated in tumor malignancy, which makes PRMT6 a potential target for anti‐cancer therapy.^[^ ^26^, ^27^, ^28^, ^29^, ^30^ ^]^ Further DARTS coupled with immunoblots confirmed that metformin bound and protected PRMT6 from pronase digestion in MCF7, MDA‐MB‐436, and MDA‐MB‐468 cells (Figure 1C; Figure S2A, Supporting Information). To corroborate these findings, we performed an isothermal titration calorimetry (ITC) assay with purified bacterially expressed PRMT6 and metformin. Metformin directly bound PRMT6 with an equilibrium dissociation constant of 0.20 µm (Figure 1D), which is also evidenced by microscale thermophoresis assay (Figure S2B, Supporting Information). Notably, by analyzing the mass spectrometry data for proteins immunoprecipitated from mouse embryonic fibroblast (MEF) lysates using a photoactive metformin probe by Ma et al.,^[^ ^12^ ^]^ we also found the presence of PRMT6. These results indicate that metformin directly binds to PRMT6.

We next examined whether metformin inhibits breast cancer cell proliferation by targeting PRMT6. Knockdown of *PRMT6* in MCF7 cells rendered the cells insensitive to metformin treatment (Figure 1E; Figure S2C, Supporting Information). Further immunofluorescence analysis of Ki‐67, a nuclear proliferation marker, confirmed that metformin treatment reduced the proliferation of control but not *PRMT6*‐knockdown MCF7 cells (Figure 1F). Similar results were observed in *PRMT6*‐knockdown MDA‐MB‐436 and MDA‐MB‐468 cells (Figure S2D, Supporting Information). For MDA‐MB‐231 cells that are less sensitive to metformin, high concentrations of metformin inhibited the growth of control but not *PRMT6*‐knockdown cells (Figure S2E,F, Supporting Information). As a control, knockdown of other potential metformin‐interacting proteins, including EZR, DDX39A/B, PABPC1, and OTUB1 did not abolish the inhibitory effect of metformin on MCF7 cell growth (Figure S2G, Supporting Information).

Interestingly, metformin still reduced the proliferation of *PRMT6*‐knockdown HepG2 cells (Figure S2H, Supporting Information). To understand why the sensitivity of *PRMT6*‐knockdown cells to metformin varies, we assessed PRMT6 expression in different cell lines. PRMT6 expression was higher in MDA‐MB‐436, MDA‐MB‐468, and MCF7 than in HeLa, HepG2, and MDA‐MB‐231 cells (Figure 1G). The lower PRMT6 expression in MDA‐MB‐231 than MCF7 correlates with their sensitivity to metformin (Figure S1B, Supporting Information). Intriguingly, the expression of AMPK anti‐correlates with PRMT6 expression in these cells (Figure 1G). By analyzing The Cancer Genome Atlas (TCGA), we found that the ratio of AMPK/PRMT6 expression was markedly lower in MCF7 than in other cell lines (Figure S2I, Supporting Information). Moreover, in breast cancer patients, *PRMT6* transcription was elevated and *AMPK* transcription was decreased (Figure S2J, Supporting Information).

Given the anti‐correlation between PRMT6 and AMPK, we examined whether AMPK represses PRMT6 expression. Indeed, overexpression of *AMPK* in MCF7, MDA‐MB‐436, MDA‐MB‐468, and HepG2 reduced the protein level of PRMT6 (Figure S2K, Supporting Information). Meanwhile, knockdown of *AMPK* in HepG2 increased PRMT6 expression (Figure S2K, Supporting Information), suggesting that the relatively lower PRMT6 expression in HepG2 may be related to its higher AMPK expression levels. To determine how AMPK regulates PRMT6 expression, we first examined whether AMPK directly binds to the *PRMT6* promoter to repress its transcription. However, chromatin immunoprecipitation (ChIP) analysis revealed no significant binding of AMPK to the *PRMT6* promoter region (Figure S2L, Supporting Information). We next evaluated the stability of *PRMT6* mRNA in *AMPK*‐knockdown MCF7 cells using actinomycin D treatment. The results indicated that AMPK depletion did not markedly alter the half‐life of *PRMT6* mRNA (Figure S2M, Supporting Information). In contrast, cycloheximide (CHX) chase assays showed that *AMPK* knockdown extended the half‐life of PRMT6 protein (Figure S2N, Supporting Information). These data suggest that AMPK regulates PRMT6 primarily at the post‐translational level, rather than through transcriptional repression or modulation of mRNA stability.

These above findings prompted us to examine whether inhibiting or depletion of AMPK may increase PRMT6 expression and sensitize cells to metformin treatment. We treated metformin‐insensitive MDA‐MB‐231 cells with compound C, an AMPK inhibitor, to increase PRMT6 expression followed by metformin treatment (Figure S2O, Supporting Information). The results showed that metformin synergized with compound C to reduce cell proliferation (Figure S2O, Supporting Information). Moreover, knockdown of *AMPK* in MDA‐MB‐231 cells potentiated the efficacy of metformin on growth inhibition (Figure S2P, Supporting Information).

### 2.3 Metformin Inhibits the Activity of PRMT6 to Catalyze Asymmetric Dimethylation of H3R2

**Figure 2:**
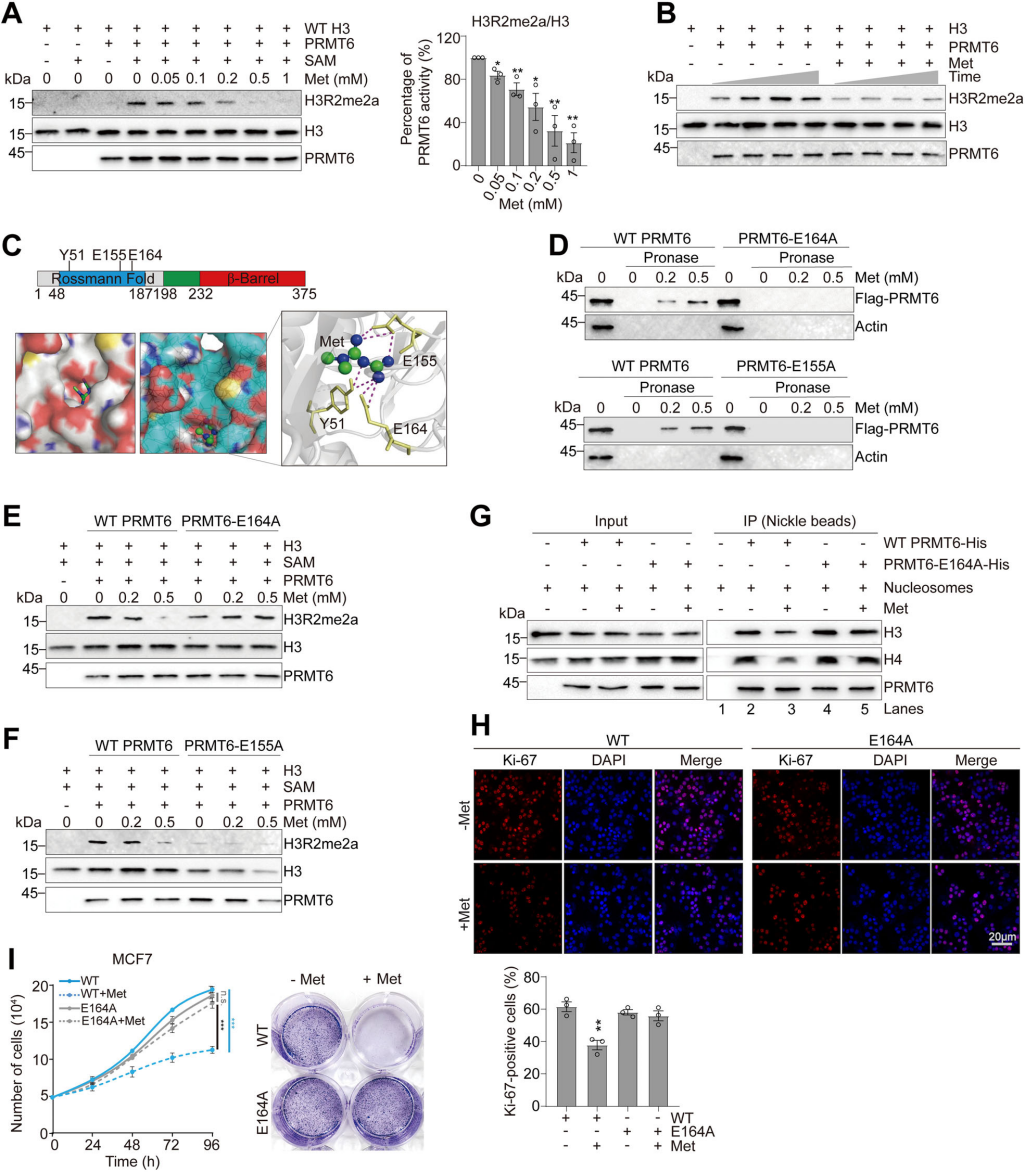
Metformin impairs breast cancer cell growth primarily by inhibiting PRMT6‐catalyzed H3R2me2a. A,B) In vitro HMT activity showing that metformin inhibited the activity of PRMT6 to catalyze H3R2me2a. 0.4 µg purified recombinant PRMT6 was incubated with 0.2 µg purified recombinant histone H3 at 37 °C and 0–1 mm metformin for 0.5 h (A) or with 0.5 mm metformin for different time (B). The activity of PRMT6 was indicated by relative intensity of H3R2me2a/H3. C) Molecular docking assay showing the binding sites of PRMT6 for metformin. D) DARTS experiments showing mutation of E164A and E155A abolished the interaction between metformin and PRMT6. The purified WT PRMT6, PRMT6‐E164A, and PRMT6‐E155A were incubated with 0–0.5 mm metformin followed by pronase digestion. E,F) In vitro HMT assay showing metformin inhibited the activity of WT PRMT6 but not PRMT6‐E164A and PRMT6‐E155A. G) Pulldown assay showing metformin (0.5 mm) reduced the binding of WT PRMT6 but not PRMT6‐E164A to nucleosomes. H,I) Ki‐67 staining, growth curves, and colony formation assay of WT and PRMT6‐E164A MCF7 cells treated with or without 1 mm metformin. For (A,H,I), data represent means ± SEM; *n* = 3 biological independent experiments; two‐tailed *t*‐tests (A,H) and two‐tailed paired *t*‐tests (I) were used for statistical analysis. * *P *< 0.05, ** *P *< 0.01, *** *P *< 0.001. For (B,D–G), shown are the typical examples of two biological independent experiments.

As PRMT6 is the primary enzyme responsible for H3R2me2a, we examined whether metformin regulates PRMT6 activity. We performed an in vitro histone methyltransferase (HMT) assay with purified bacterially expressed PRMT6 and histones in the presence or absence of metformin. The HMT assay showed that metformin repressed PRMT6 activity in a dose‐ and time‐dependent manner (Figure **2**A,B). A similar inhibitory effect was observed for PRMT6 when in vitro assembled octamers and nucleosomes were used as the substrates (Figure S3A,B, Supporting Information). In contrast, metformin exhibited minimal inhibitory activity against other histone methyltransferases within the PRMT family, such as PRMT1, PRMT4, PRMT5, and PRMT7 (Figure S3C,D, Supporting Information), indicating its selectivity for PRMT6.

To map the region(s) of PRMT6 responsible for interacting with metformin, we generated a series of truncated PRMT6 mutants and evaluated their binding using the DARTS assay. Metformin specifically bound to full‐length PRMT6 (WT PRMT6) and a C‐terminal truncated variant (PRMT6‐ΔC), but showed no detectable binding to either the N‐terminal (PRMT6‐N) or C‐terminal (PRMT6‐C) fragments (Figure S3E, Supporting Information), suggesting that the central SAMD domain is essential for metformin binding. This was confirmed by DARTS assay using the purified SAMD domain, which demonstrated direct interaction with metformin (Figure S3F, Supporting Information). Molecular docking analysis further indicated that residues E155 to E164 within the SAMD domain are critical for metformin binding (Figure S3F, Supporting Information). Consistent with this, deletion of residues 150–170 (PRMT6‐D_150‐170_) completely abolished metformin binding (Figure S3F, Supporting Information). Molecular docking analysis of full‐length PRMT6 with metformin indicated that residues E155 and E164 within PRMT6 are involved in its binding to metformin (Figure 2C). In addition, Y51 was also identified as a key residue mediating the interaction with metformin (Figure 2C). PRMT6 Y51, E155, and E164 showed a high degree of conservation in a wide range of organisms (Figure S3G, Supporting Information). Moreover, mutation of PRTM6‐Y51F and E155Q was found in cancer patients. We thus individually mutated these three residues within PRMT6 (Y51F, E155A, E155Q, E164A). Mutation of these three PRMT6 residues abolished the interaction between metformin and PRMT6 (Figure 2D; Figure S3H,I, Supporting Information). Moreover, mutation of these residues abrogated the inhibitory effect of metformin on PRMT6‐catalyzed H3R2me2a (Figure 2E,F; Figure S3J, Supporting Information). As the Y51F, E155A, and E155Q mutations largely compromised the HMT activity of PRMT6 (Figure 2F; Figure S3J, Supporting Information), we thus focused on the PRMT6‐E164A mutant.

By analyzing the catalogue of somatic mutations in cancer patients, we identified 2 additional mutations around PRMT6 E164, including L162M and S165P. L162M is derived from gastric cancer, while S165P is derived from esophageal cancer. We thus constructed these two PRMT6 mutants and performed the DARTS assay to examine their binding to metformin. Although mutation of L162M had no effect on metformin‐PRMT6 interaction, mutation of S165P abrogated the interaction between metformin and PRMT6 (Figure S3H, Supporting Information). Consistently, mutation of S165P abolished the inhibitory effect of metformin on PRMT6 HMT activity (Figure S3K, Supporting Information). These results suggest that patients harboring the PRMT6‐S165P mutant may be less sensitive to metformin treatment.

To understand how metformin inhibits the enzymatic activity of PRMT6, we examined the effect of metformin on the binding of PRMT6 to its histone substrate. We performed a pulldown assay with purified PRTM6 and in vitro assembled nucleosomes. Metformin inhibited the binding of WT PRMT6 but not PRMT6‐E164A to nucleosomes (Figure 2G, lane 2 vs lane 3, lane 4 vs lane 5), suggesting that metformin interferes with the binding of PRMT6 to nucleosomes.

We next used CRISPR‐Cas9 genome‐editing technology to construct knock‐in expression of PRMT6‐E164A in MCF7 cells (Figure S3L, Supporting Information). Mutation of PRMT6‐E164A blunted the inhibitory role of metformin on H3R2me2a, Ki‐67 expression, and growth of MCF7 cells (Figure 2H,I; Figure S3M,N, Supporting Information). A similar result was observed for PRMT6‐E164A MDA‐MB‐468 cells (Figure S3O, Supporting Information).

To exclude potential off‐target or cytotoxic effects at the metformin concentration used, which might otherwise confound the interpretation of its anti‐proliferative role, we evaluated its influence on cell death pathways, including necrosis and apoptosis. Our data showed that the concentration of metformin employed did not significantly induce necrosis or apoptosis in either WT MCF7 or PRMT6‐E164A MCF7 cells (Figure S4A, Supporting Information). We further examined whether metformin could synergize with established PRMT6 inhibitors (MS049, EPZ020411) in suppressing MCF7 cell growth. Both inhibitors effectively inhibited MCF7 growth in a PRMT6‐dependent manner (Figure S4B, Supporting Information), confirming their on‐target activity. Metformin produced a slightly additive or synergistic effect when combined with these inhibitors (Figure S4C,D, Supporting Information). These results support the conclusion that metformin suppresses breast cancer cell growth primarily through specific inhibition of PRMT6, rather than through non‐specific cytotoxicity or synergistic modulation of other PRMT6‐related pathways.

### 2.4 Metformin Inhibits PRMT6‐Catalyzed H3R2me2a to Repress *PRMT6* Transcription

**Figure 3:**
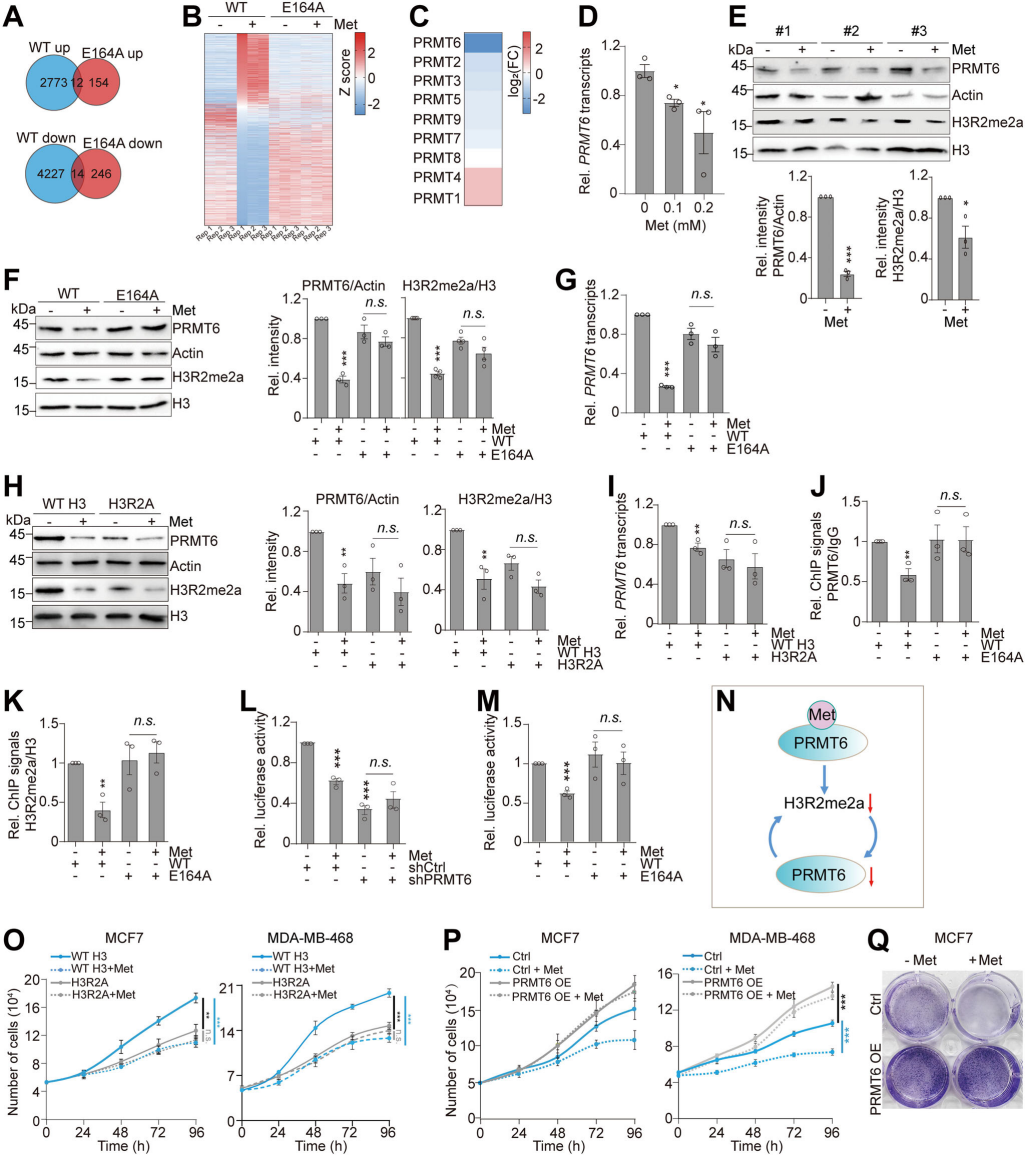
Metformin inhibits PRMT6‐catalyzed H3R2me2a to repress *PRMT6* transcription. A,B) Venn diagram and heatmap showing genes regulated by 1 mm metformin (Met) for 48 h in WT PRMT6 and PRMT6‐E164A mutant by RNA‐seq (Log_2_(Fold change) ≥ 1, log_2_(Fold change) ≤ −1, *P *≤ 0.05). C) RNA‐seq analysis of the effect of 1 mm metformin on the transcription of PRMT family members. D) RT‐qPCR analysis of the effect of 0–0.2 mm metformin on *PRMT6* transcription. E) Immunoblot analysis of the effect of metformin (60 mg kg^−1^) on the levels of PRMT6 and H3R2me2a in MMTV transgenic breast tumors. F) Immunoblot analysis of PRMT6 and H3R2me2a in WT and PRMT6‐E164A MCF7 cells when treated with 0.5 mm metformin for 48 h. G) RT‐qPCR analysis of *PRMT6* transcription in WT and PRMT6‐E164A MCF7 cells when treated with 0.5 mm metformin for 48 h. H) Immunoblot analysis of the effect of 0.5 mm metformin on the levels of PRMT6 and H3R2me2a in MCF7 cells stably expressing WT H3 and H3R2A mutant for 48 h. I) RT‐qPCR analysis of the effect of 0.5 mm metformin on *PRMT6* transcription in MCF7 cells stably expressing WT H3 and H3R2A mutant for 48 h. J,K) ChIP‐qPCR analysis of the enrichment of PRMT6 and H3R2me2a/H3 at the *PRMT6* promoter in WT and PRMT6‐E164A MCF7 cells treated with or without 0.5 mm metformin for 48 h. L,M) Luciferase reporter assay for PRMT6 in control (shCtrl), *PRMT6*‐knockdown (shPRMT6), WT, and PRMT6‐E164A MCF7 cells treated with or without 0.5 mm metformin for 48 h. N) Diagram showing metformin inhibits the feed‐forward loop to reduce H3R2me2a and repress *PRMT6* transcription. O) Analysis of the effect of 1 mm metformin on the growth of MCF7 and MDA‐MB‐468 cells stably expressing WT H3 and H3R2A mutant. P,Q) Analysis of the effect of 1 mm metformin on growth and colony formation of control (Ctrl) and *PRMT6*‐overexpression (PRMT6 OE) MCF7 and MDA‐MB‐468 cells. For (D–M,O,P), data represent means ± SEM; *n* = 3 biological independent experiments; two‐tailed *t*‐tests (D–M) and two‐tailed paired *t*‐tests (O,P) were used for statistical analysis. * *P *< 0.05, ** *P *< 0.01, *** *P *< 0.001, *n.s*., no significance. For (Q), shown are the typical examples of two biological independent experiments.

To determine whether metformin regulates the transcriptome of breast cancer cells by binding and inhibiting PRMT6, we treated WT and PRMT6‐E164A mutants with metformin and then analyzed their transcriptomes by RNA‐seq. Metformin significantly down‐regulated the transcription of 4241 genes and up‐regulated the transcription of 2785 genes in WT cells (Figure S5A, Supporting Information), whereas these genes were largely unaffected by metformin in PRMT6‐E164A mutant (**Figure** **3**A,B), suggesting that metformin regulates gene expression primarily by binding and inhibiting PRMT6 in MCF7 cells. Kyoto Encyclopedia of Genes and Genomes (KEGG) pathway analysis revealed that metformin‐repressed genes are enriched in pathways, including homologous recombination, cell cycle, homologous recombination, DNA replication, etc. (Figure S5B, Supporting Information).

Strikingly, we found that metformin markedly repressed the transcription of *PRMT6* among 9 PRMT family members (Figure 3C), which was confirmed by quantitative reverse transcription PCR (RT‐qPCR) (Figure 3D; Figure S5C, Supporting Information). Consistently, metformin reduced the levels of PRMT6 and H3R2me2a in MCF7, MDA‐MB‐436, and MDA‐MB‐468 cells but not in MD‐MBA‐231 and HeLa cells (Figure S5D–F, Supporting Information), which is consistent with their growth sensitivity to metformin (Figure S1B, Supporting Information). Metformin has been reported to reduce histone H3K27me3 and H3K4me3 in prostate organoids and *Caenorhabditis elegans*, respectively.^[^ ^22^, ^23^ ^]^ However, none of these histone modifications were affected by metformin in breast cancer cells (Figure S5D, Supporting Information), suggesting the effect of metformin depends on cell and tissue types. In addition, metformin reduced the levels of PRMT6 and H3R2me2a in both breast cancer mouse xenograft and MMTV transgenic breast tumors (Figure 3E; Figure S5G, Supporting Information). Meanwhile, metformin reduced the levels of PRMT6 and H3R2me2a in WT but not in the PRMT6‐E164A mutant, and this regulation occurred at the transcriptional level (Figure 3F,G).

We next aimed to understand how metformin represses *PRMT6* transcription. Knockdown of *AMPK* did not restore metformin‐induced PRMT6 reduction in MCF7 cells (Figure S6A, Supporting Information). Moreover, metformin repressed PRMT6 expression independent on DNA methylation as treatment with 5‐Azacytidine (5‐AzaC), a nucleoside analog of cytidine that inhibits DNA methylation, did not rescue the declined PRMT6 in metformin‐treated MCF7 cells (Figure S6B, Supporting Information). Since metformin directly inhibits PRMT6‐catalyzed H3R2me2a, we wondered whether metformin repressed *PRMT6* transcription by reducing H3R2me2a. MCF7 cells were transfected with H3R2A overexpression plasmid to reduce global H3R2me2a, which is based on the observation that H3R2A mutant acted *in trans* to inhibit the activity of PRMT6 to methylate WT H3 but had no effect on the activity of other histone methyltransferases, such as PRMT4 (Figure S6C,D, Supporting Information). Indeed, the global level of H3R2me2a was specifically reduced in H3R2A‐overexpressing cells (Figure 3H; Figure S6E, Supporting Information). The expression of PRMT6 was markedly reduced and less sensitive to metformin treatment in H3R2A mutant (Figure 3H,I; Figure S6E,F, Supporting Information). Similar results were observed in MDA‐MB‐468 cells that overexpress H3R2A (Figure S6G,H, Supporting Information).

To directly examine whether PRMT6 promotes itself transcription via H3R2me2a, we examined the effect of metformin on the occupancy of PRMT6 and H3R2me2a at the *PRMT6* promoter region by ChIP. Metformin reduced the occupancy of PRMT6 and H3R2me2a at the *PRMT6* promoter region in WT but not in the PRMT6‐E164A mutant (Figure 3J,K). We also cloned the *PRMT6* promoter region into a pGL3 luciferase reporter vector. Overexpression of *PRMT6* significantly increased *PRMT6* promoter‐driven luciferase activity, while knockdown of *PRMT6* or mutation of H3R2A significantly reduced *PRMT6* promoter‐driven luciferase activity (Figure S6I, Supporting Information), indicating that PRMT6‐catalyzed H3R2me2a promotes *PRMT6* transcription. Moreover, *PRMT6* promoter‐driven luciferase activity was less sensitive to metformin treatment in both *PRMT6*‐knockdown and PRMT6‐E164A cells (Figure 3L,M). These results revealed a feed‐forward loop to amplify PRMT6 expression and H3R2me2a, and metformin inhibited this loop to reduce H3R2me2a (Figure 3N): metformin directly binds PRMT6 and inhibits its activity to catalyze H3R2me2a, leading to decreased H3R2me2a enrichment at the *PRMT6* promoter and reduced *PRMT6* transcription, which further decreases H3R2me2a.

To delineate the H3R2me2a‐responsive elements within the PRMT6 promoter, we conducted promoter truncation analysis using a dual‐luciferase reporter assay. Deletion of the ‐1500 to ‐1000 region resulted in a marked decrease in luciferase activity and abolished metformin's inhibitory effect on fluorescence (Figure S6J, Supporting Information). To further identify the potential transcription factors that bind to this region to activate *PRMT6* transcription, we screened the JASPAR database, which identified SPZ1(spermatogenic leucine zipper 1) and RFX6 (regulatory factor X6) as the top candidates. Knockdown of *SPZ1*, but not *RFX6*, significantly reduced *PRMT6* transcription (Figure S6K,L, Supporting Information). ChIP‐qPCR analysis further demonstrated that metformin treatment diminished SPZ1 enrichment at the *PRMT6* promoter (Figure S6M, Supporting Information). These results indicate that SPZ1 is a key transcription factor that activates *PRMT6* transcription.

Next, we examined the effect of metformin on the proliferation of breast cancer cells stably overexpressing WT H3 and H3R2A mutant. Expression of H3R2A mutant in MCF7 and MDA‐MB‐468 cells attenuated the inhibitory effect of metformin on cell proliferation (Figure 3O; Figure S6N, Supporting Information). Moreover, overexpression of *PRMT6*, but not other PRMT family members, alleviated the inhibitory effect of metformin on cell proliferation (Figure 3P,Q; Figure S6O–V, Supporting Information). Collectively, these studies indicate that metformin inhibits PRMT6‐catalyzed H3R2me2a to reduce PRMT6 expression and impair cell proliferation.

### 2.5 Metformin Inhibits PRMT6‐H3R2me2a Axis and MCF7 Cell Growth Independent on SAM/SAH Metabolism

Metformin is known to reduce the synthesis of SAH, an inhibitor of SAM‐dependent methyltransferases, while simultaneously promoting SAM accumulation, thereby elevating the cellular SAM/SAH ratio.^[^ ^21^ ^]^ Consistent with this, metformin treatment led to a significant increase in the SAM/SAH ratio in both WT and PRMT6‐E164A mutant cells (Figure S7A, Supporting Information), indicating that metformin modulates SAM/SAH metabolism independent of PRMT6. To determine whether metformin affects the PRMT6–H3R2me2a axis through metabolic regulation, we repeated key experiments under glucose‐free conditions to decrease SAM/SAH levels and minimize AMPK‐mediated metabolic effects. Metformin continued to increase the SAM/SAH ratio even in the absence of glucose (Figure S7B, Supporting Information). Immunoblot analysis confirmed that metformin still reduced both PRMT6 protein and H3R2me2a levels under glucose deprivation (Figure S7C, Supporting Information). Furthermore, ChIP‐qPCR revealed sustained decreases in PRMT6 binding and H3R2me2a enrichment at the *PRMT6* promoter (Figure S7D, Supporting Information). Notably, the reduction in H3R2me2a induced by metformin was completely abolished in PRMT6‐E164A mutant cells under these conditions (Figure S7E, Supporting Information), indicating that metformin inhibited PRMT6‐catalyzed H3R2me2a independent of glucose metabolism. To further validate that metformin's effects on the PRMT6–H3R2me2a axis and cell proliferation are independent of metabolic changes, we treated MCF7 cells with SAH to experimentally lower the SAM/SAH ratio (Figure S7F, Supporting Information). Metformin still reduced PRMT6 expression and H3R2me2a levels in SAH‐treated cells (Figure S7G, Supporting Information) and continued to inhibit cell proliferation (Figure S7H, Supporting Information). Additionally, we knocked down serine hydroxymethyltransferase 2 (*SHMT2*), which has been reported to decrease intracellular SAM/SAH.^[^ ^21^ ^]^ Metformin repressed the growth of *SHMT2‐*knockdown cells, whereas PRMT6‐E164A mutation abolished this effect (Figure S7I, Supporting Information). Collectively, these results demonstrate that metformin inhibits breast cancer cell proliferation through direct targeting of PRMT6, rather than via indirect metabolic mechanisms.

### 2.6 Metformin Increases DNA Methylation Primarily by Inhibiting PRMT6‐Catalyzed H3R2me2a

**Figure 4:**
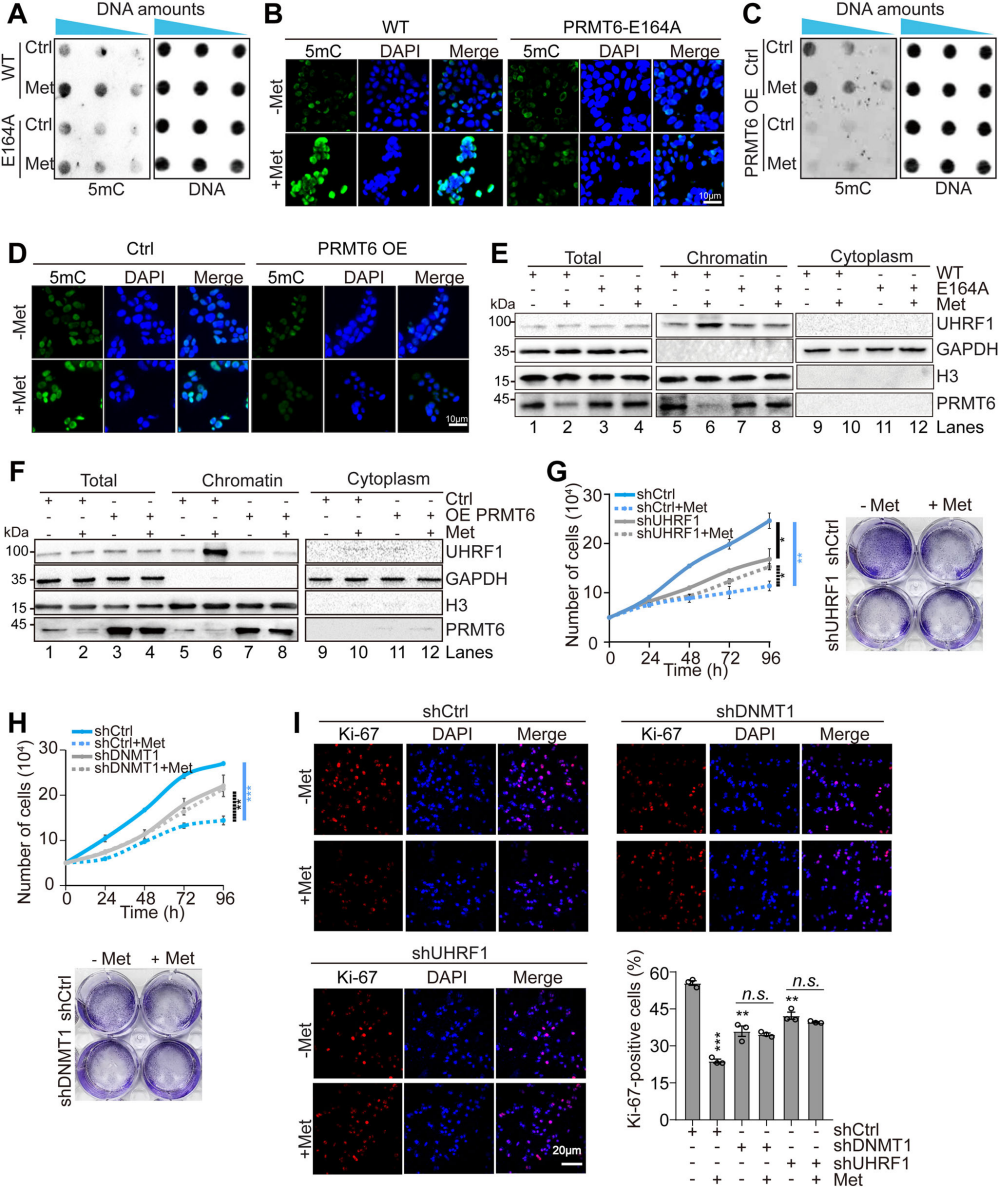
Metformin promotes DNA methylation primarily by inhibiting PRMT6‐catalyzed H3R2me2a. A) Dot blot analysis of DNA methylation (5mC) in WT and PRMT6‐E164A MCF7 cells treated with or without 0.5 mm metformin for 48 h. The triangle symbol indicates the varying amounts of genomic DNA loaded. B) Immunofluorescence analysis of the effect of 0.5 mm metformin on DNA methylation in WT and PRMT6‐E164A MCF7 cells for 48 h. C) Dot blot analysis of the effect of 0.5 mm metformin on DNA methylation in control (Ctrl) and *PRMT6*‐overexpressing (PRMT6 OE) MCF7 cells for 48 h. D) Immunofluorescence analysis of the effect of 0.5 mm metformin on DNA methylation in control (Ctrl) and *PRMT6*‐overexpressing (PRMT6 OE) MCF7 cells for 48 h. E) Subcellular fractionation assay showing 0.5 mm metformin induced the association of UHRF1 with chromatin in WT but not in the PRMT6‐E164A MCF7 mutant. F) Subcellular fractionation assay showing overexpression of PRMT6 abolished the inducing effect of metformin (0.5 mm) on chromatin association of UHRF1 in MCF7 cells. G,H) Growth curves and colony formation assay of control (shCtrl), *DNMT1*‐knockdown (shDNMT1), and *UHRF1*‐knockdown (shUHRF1) MCF7 cells treated with or without 1 mm metformin. I) Ki‐67 staining of control (shCtrl), *DNMT1*‐knockdown (shDNMT1), and *UHRF1*‐knockdown (shUHRF1) MCF7 cells treated with or without 1 mM metformin. For (G–I), data represent means ± SEM; *n* = 3 biological independent experiments; two‐tailed *t*‐tests (I) and two‐tailed paired *t*‐test (G,H) were used for statistical analysis. * *P *< 0.05, ** *P *< 0.01, *** *P *< 0.001, *n.s*., no significance. For (A–F), shown are the typical examples of three biological independent experiments.

Having established PRMT6 as the primary target of metformin in breast cancer cells, we aimed to identify the downstream events of PRMT6. Tumor cells generally exhibit global hypomethylation and loci‐specific hypermethylation, leading to genomic instability and silencing of tumor suppressors, respectively.^[^ ^34^ ^]^ PRMT6‐catalyzed H3R2me2a has been reported to reduce DNA methylation (5‐methylcytosine, 5mC) by negatively regulating the association of UHRF1 (ubiquitin‐like containing PHD ring finger 1) with chromatin.^[^ ^20^ ^]^ As metformin inhibits PRMT6‐catalyzed H3R2me2a, we surmised that metformin may relieve the inhibitory effect of PRMT6‐catalyzed H3R2me2a on DNA methylation. In support of this hypothesis, metformin treatment increased global DNA methylation as determined by dot blots and immunofluorescence with anti‐5mC antibody (Figure S8A,B, Supporting Information). Metformin had no effect on DNA methylation in *PRMT6*‐knockdown or H3R2A‐overexpressing cells (Figure S8B–F, Supporting Information). In addition, mutation of PRMT6‐E164A or overexpression of *PRMT6* abolished metformin‐induced increase of DNA methylation (**Figure** **4**A–D). These data indicate that metformin induces DNA methylation primarily by inhibiting PRTM6‐catalyzed H3R2me2a.

DNA methylation is established by *de novo* DNA methyltransferases DNMT3A and DNMT3B and maintained primarily by the maintenance enzyme DNMT1. DNMT1 is recruited to hemi‐methylated DNA with the aid of UHRF1.^[^ ^35^, ^36^ ^]^ The association of UHRF1 with chromatin is negatively regulated by PRMT6‐catalyzed H3R2me2a.^[^ ^20^ ^]^ To examine the effect of metformin on the binding of UHRF1 to chromatin, we performed a nuclear fractionation assay of MCF7 cells treated with or without metformin. Metformin substantially enhanced the association of UHRF1 at chromatin without affecting its expression (Figure S8G, Supporting Information). In contrast, metformin had no effect on UHRF1 binding at chromatin in the PRMT6‐E164A mutant (Figure 4E, lane 7 vs lane 8). Similarly, overexpression of *PRMT6* abrogated metformin‐induced binding of UHRF1 at chromatin (Figure 4F, lane 7 vs lane 8). Moreover, knockdown of *UHRF1* or *DNMT1* ameliorated metformin‐induced increase of DNA methylation (Figure S8H,I, Supporting Information). Knockdown of *UHRF1* or *DNMT1* abolished the inhibitory effect of metformin on Ki‐67 expression and cell growth (Figure 4G‐I; Figure S8J,K, Supporting Information). Together, these data suggest that metformin inhibits PRMT6‐catalyzed H3R2me2a, which then enhances the association of UHRF1 with chromatin to promote DNMT1‐catalyzed DNA methylation.

### 2.7 Metformin Regulates the Transcription of DNA Replication‐Associated Genes Primarily by Regulating the PRMT6‐H3R2me2a‐5mC Axis

**Figure 5:**
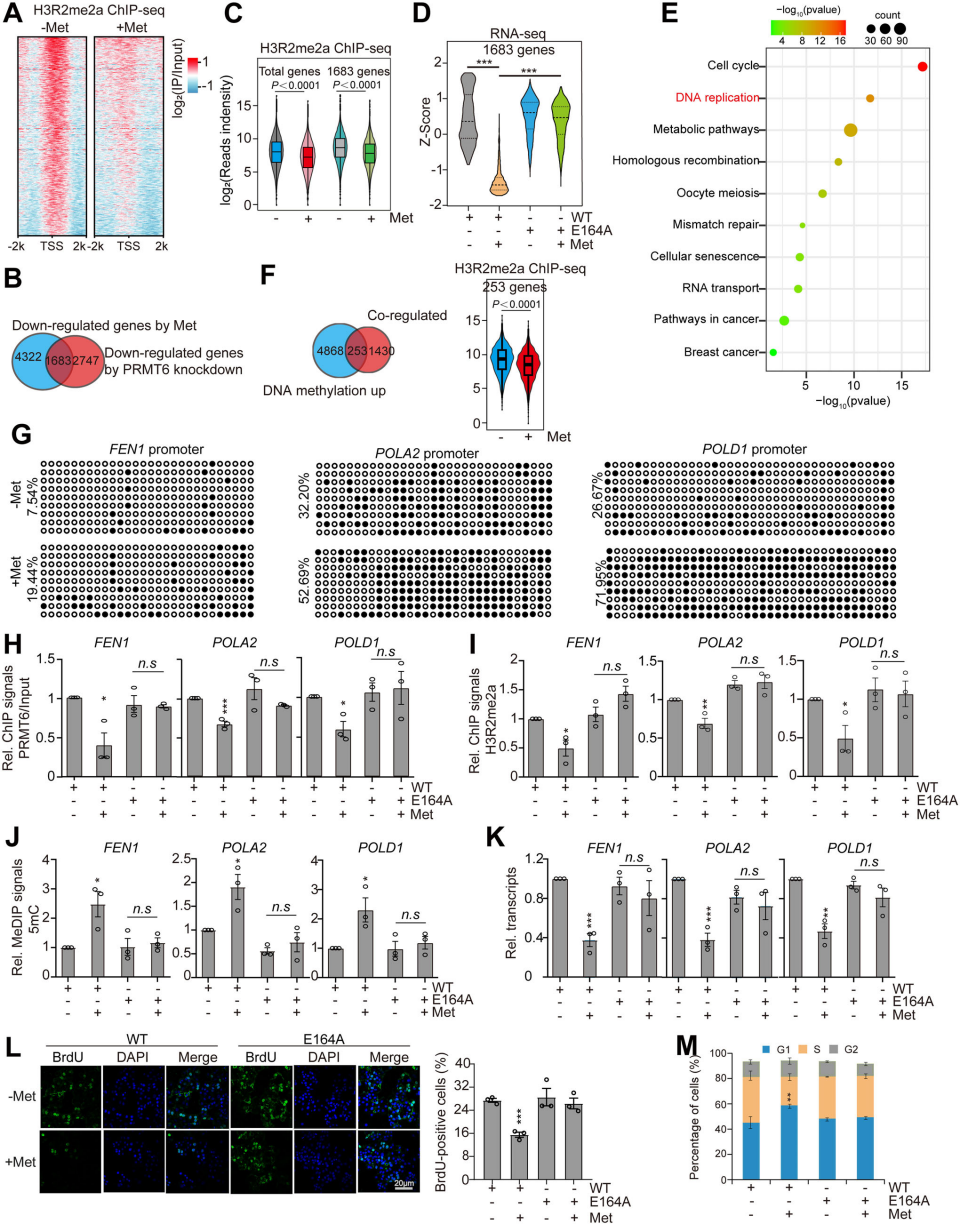
Metformin regulates DNA replication‐related gene expression via the PRMT6‐H3R2me2a‐5mC axis. A) Heatmap showing the genome‐wide enrichment of H3R2me2a at TSS +/− 2kb regions in MCF7 cells treated with or without 1 mm metformin for 48 h. Color‐scaled intensities are in units of log_2_ (IP/Input). B) Venn diagrams showing the overlap of 1683 genes that were down‐regulated (Fold change ≤ 0.75, *P *≤ 0.05) by metformin and *PRMT6* knockdown. C) Box plots showing that the occupancy of H3R2me2a at the promoter regions of the indicated genes were significantly reduced by 1 mm metformin in MCF7 cells. D) Box plots showing transcriptional changes of indicated genes in WT and PRMT6‐E164A MCF7 cells when treated with 1 mm metformin. Centre lines denote medians, box limits denote 25th and 75th percentiles, and whiskers denote maximum and minimum values. Two‐tailed *t*‐test was used for statistical analysis. E) KEGG analysis of 1683 genes co‐regulated by metformin and *PRMT6* knockdown. F) Left panel: Venn diagram illustrating the overlap between 1683 genes co‐regulated by metformin and PRMT6, and genes exhibiting increased promoter DNA methylation. Right panel: Box plots demonstrating a significant reduction in H3R2me2a occupancy at the promoter regions of indicated genes following treatment with 1 mm metformin in MCF7 cells. G) Bisulfite sequencing analysis of 5mC at the promoter regions of *FEN1*, *POLA2*, and *POLD1* in MCF7 cells treated with or without 1 mm metformin for 48 h. Open circles, unmethylated CpG; filled circles, methylated CpGs. H,I) ChIP‐qPCR analysis of PRMT6 and H3R2me2a/H3 occupancy at the promoter regions of *FEN1*, *POLA2*, and *POLD1* in WT and PRMT6‐E164A MCF7 cells treated with or without 1 mm metformin for 48 h. J) MeDIP analysis of 5 mC at promoter regions of *FEN1*, *POLA2*, and *POLD1* in WT and PRMT6‐E164A MCF7 cells treated with or without 1 mm metformin for 48 h. K) RT‐qPCR analysis of the transcription of *FEN1*, *POLA2*, and *POLD1* in WT and PRMT6‐E164A MCF7 mutant treated with or without 0.5 mm metformin for 48 h. L) BrdU staining analysis of WT and PRMT6‐E164A MCF7 cells treated with or without 1 mm metformin for 48 h. M) Analysis of cell cycle progression in WT and PRMT6‐E164A MCF7 cells treated with or without 1 mm metformin for 48 h. For (H–M), data represent means ± SEM; *n* = 3 biological independent experiments; two‐tailed *t*‐tests were used for statistical analysis. * *P *< 0.05, ** *P *< 0.01, *** *P *< 0.001, *n.s*., no significance.

We next performed ChIP with high‐throughput sequencing (ChIP‐seq) for H3R2me2a in MCF7 cells when treated with metformin. Metformin reduced the occupancy of H3R2me2a at transcription start sites (TSS) (**Figure** **5**A), which is consistent with the enrichment of H3R2me2a at the TSS of active genes.^[^ ^37^ ^]^ We also performed RNA‐seq for metformin‐treated and *PRMT6*‐knockdown MCF7 cells, among which the transcription of 1683 genes was both significantly down‐regulated by metformin and *PRMT6* knockdown (Figure 5B). Moreover, metformin reduced the occupancy of H3R2me2a at the TSS of these 1683 genes (Figure 5C). Mutation of PRMT6‐E164A abolished the inhibitory effect of metformin on their transcription (Figure 5D), suggesting that metformin inhibits PRMT6 activity to repress the transcription of these 1683 genes.

KEGG pathway analysis revealed that the above 1683 genes were enriched in cell cycle, DNA replication, metabolic pathways, homologous recombination, etc. (Figure 5E). Whole genome methylation sequencing revealed that among these 1683 genes, 253 genes had increased DNA methylation in their promoters under metformin treatment (Figure 5F). The integrative analysis with H3R2me2 ChIP‐seq data revealed that these 253 genes have reduced H3R2me2a occupancy under metformin treatment (Figure 5F), suggesting that metformin reduced H3R2me2a at these gene promoters, leading to increased DNA methylation and reduced gene transcription. KEGG analysis revealed that these 253 genes were significantly enriched in DNA replication (i.e., *FEN1*, *POLA2*, *POLD1*) and cell cycle (i.e., *PKMYT1*, *CDKN2C*) pathways (Figure S9A, Supporting Information). Bisulfite sequencing revealed that DNA methylation at the promoter regions of these genes was increased by metformin (Figure 5G; Figure S9B, Supporting Information).

Analysis of the whole blood transcriptome data for type 2 diabetes patients administrated with metformin for three months revealed that the transcription of *PRMT6*, *POLA2*, *POLD1*, and *FEN1* in their blood samples was reduced by metformin (Figure S9C, Supporting Information). By analyzing the TCGA database, we found that in breast cancer patients, the transcription of *POLA2*, *POLD1*, and *FEN1* was increased and positively correlated with *PRMT6* (Figure S9D,E, Supporting Information), whereas the DNA methylation of *POLD1* and *FEN1* promoters was reduced (Figure S9F, Supporting Information). This anti‐correlation implied that their transcription may be regulated by DNA methylation. Treatment with 5‐AzaC restored the reduced transcription of these three genes by metformin (Figures S9G, Supporting Information). ChIP‐qPCR revealed that metformin significantly reduced the occupancy of PRMT6 and H3R2me2a at these three genes in WT but not in the PRMT6‐E164A mutant (Figure 5H,I). Methylated‐DNA immunoprecipitation (MeDIP) analysis with anti‐5mC antibody confirmed that metformin significantly increased DNA methylation at these three genes in WT but not in the PRMT6‐E164A mutant (Figure 5J). Overexpression of *PRMT6* abolished the metformin‐induced increase of DNA methylation at these three genes (Figure S9H, Supporting Information). Consistent with the observed DNA methylation changes, metformin repressed the transcription of these three genes in WT but not in the PRMT6‐E164A mutant or *PRMT6*‐overexpressing (PRMT6 OE) cells (Figure 5K; Figure S9I, Supporting Information). Knockdown of *PRMT6* also alleviated the inhibitory effect of metformin on transcription of these three genes in both MCF7, MDA‐MB‐436, and MDA‐MB‐468 cells (Figure S9J–L, Supporting Information). Furthermore, knockdown of *UHRF1* or *DNMT1* alleviated the inhibitory effect of metformin on transcription of these three genes (Figure S10A,B, Supporting Information).

To examine the effect of metformin on DNA replication, we performed the 5‐Bromo‐ and 5‐Iododeoxyuridine (BrdU) assay to detect DNA replication.^[^ ^38^ ^]^ These results showed that metformin significantly inhibited DNA synthesis in WT but not in the PRMT6‐E164A mutant (Figure 5L). In addition, metformin treatment arrested WT cells but not PRMT6‐E164A mutant at G1/S phase (Figure 5M). As the association of UHRF1 with chromatin is coupled to DNA replication,^[^ ^35^ ^]^ we also performed the kinetic analysis of the association of UHRF1 with chromatin, DNA methylation, transcription of DNA replication‐associated genes, and BrdU incorporation when MCF7 cells were treated with metformin for 0–48 h. The results showed that metformin increased the enrichment of UHRF1 and 5mC at three DNA replication‐associated genes as early as 12 h and substantially increased at 24 h in WT but not in PRMT6‐E164A MCF7 mutant (Figure S10C,D, Supporting Information). The transcription of these three genes and BrdU incorporation were dramatically reduced by metformin at 48 h in MCF7 cells (Figure S10E,F, Supporting Information). Metformin also reduced the growth of MCF7 cells at 48 h (Figure S1A, Supporting Information). Hence, metformin promotes UHRF1 binding and DNA methylation, thus inhibiting DNA replication and cell growth in a temporal and sequential manner.

We then examined the effect of metformin in combination with the DNA replication inhibitor, hydroxyurea (HU), on cell growth. Metformin and HU synergistically impaired the growth of WT but not PRMT6‐E164A MCF7 cells (Figure S10G, Supporting Information). Metformin and HU synergistically inhibited the growth of other breast cancer cell lines, including MDA‐MB‐436 and MDA‐MB‐468 cells (Figure S10H, Supporting Information). Knockdown of *FEN1*, *POLA2*, and *POLD1* in MCF7 cells made these cells grow more severely when treated with metformin (Figure S10I, Supporting Information). Collectively, these data indicate that metformin represses the transcription of DNA replication‐associated genes and cell growth via the PRMT6‐H3R2me2a‐5mC axis.

### 2.8 Metformin Impedes Tumorigenesis Primarily by Inhibiting PRMT6‐Catalyzed H3R2me2a

We were keen to find out whether metformin has an inhibitory effect on the growth of breast cancer cells via the PRMT6‐H3R2me2a‐5mC axis in vivo. MCF7 cells were subcutaneously injected into nude mice. After tumors had developed, the tumor‐bearing mice were administered with 60 mg kg^−1^ metformin, 50 mg kg^−1^ PRMT6 inhibitor MS049, or together. Metformin and MS049 inhibited the growth of xenograft tumors (Figure S11A–C, Supporting Information). Like the results with the cell proliferation assay (Figure S4C,D, Supporting Information), metformin did not largely enhance the inhibitory effect of MS049 on tumor growth (Figure S11A–C, Supporting Information). Administration of DNMT1 inhibitor 5‐AzaC did not attenuate the inhibitory effect of metformin (Figure S11A–C, Supporting Information), which may be due to the complex effects of 5‐AzaC.^[^ ^39^ ^]^ We then performed xenograft experiments to examine the effect of metformin on the growth of MDA‐MB‐468 cells with or without *PRMT6* knockdown (shPRMT6). Metformin significantly impaired the growth of xenograft tumors derived from WT but not shPRMT6 cells (Figure S11D‐F, Supporting Information). Metformin also significantly reduced PRMT6 and H3R2me2a in WT but not in the shPRMT6 tumors (Figure S11G,H, Supporting Information). RT‐qPCR analysis revealed that the transcription of *FEN1*, *POLA2*, and *POLD1* was significantly reduced by metformin in control but not in the shPRMT6 tumors (Figure S11I, Supporting Information).

**Figure 6:**
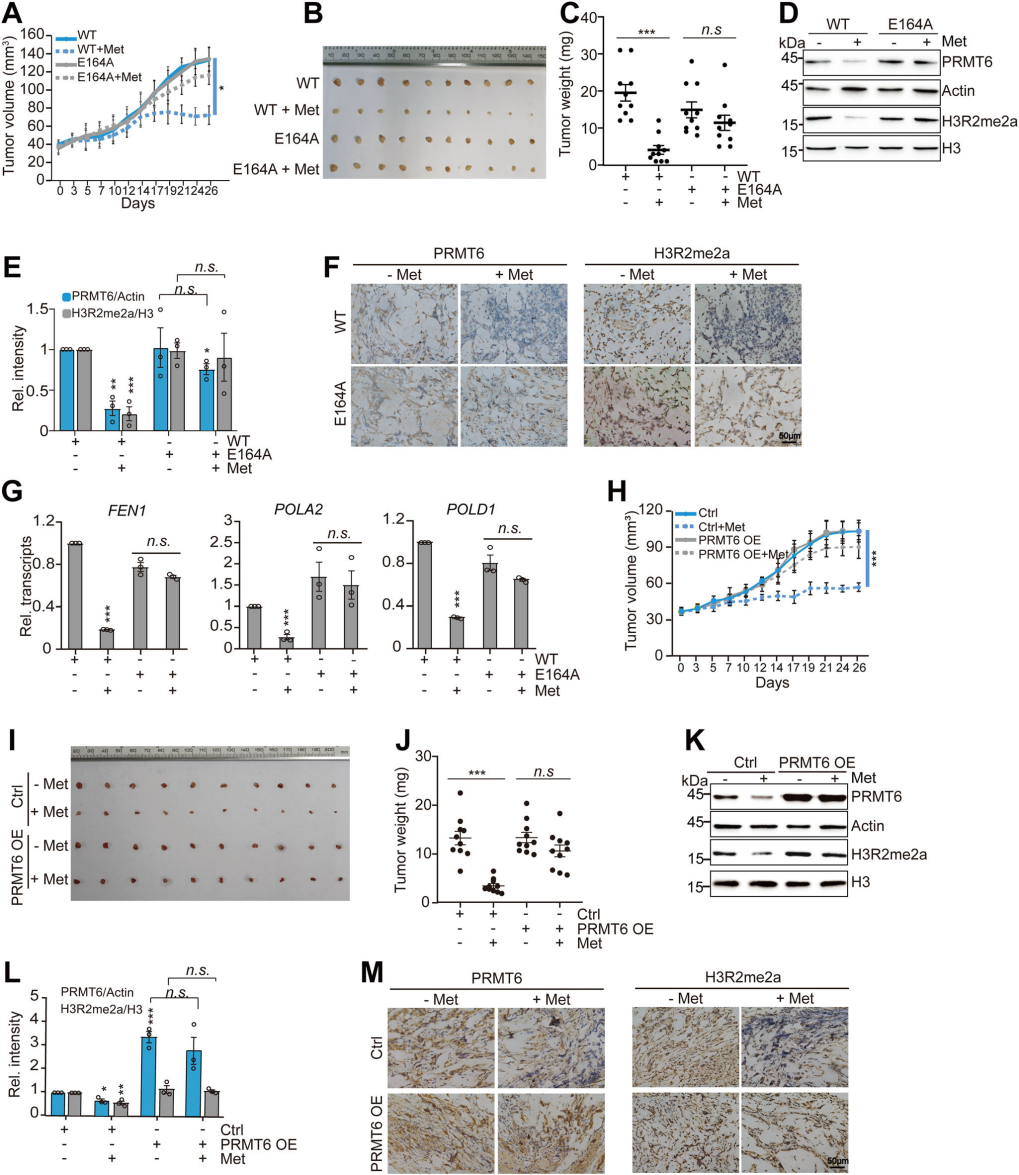
Metformin represses tumorigenesis primarily by inhibiting PRMT6‐catalyzed H3R2me2a. A–C) Metformin inhibited xenograft growth of WT but not PRMT6‐E164A MCF7 cells. The BALB/c nude mice were subcutaneously injected with MCF7 cells and received PBS (−Met) or 60 mg kg^−1^ metformin (+Met) by oral gavage (10 mice group^−1^). The xenograft tumors were measured over time and dissected at the endpoint. Quantification of the average volume of tumors over time was shown in (A). The dissected tumors and quantification of the tumor weight were shown in (B,C). D,E) Immunoblots of PRMT6 and H3R2me2a in WT and PRMT6‐E164A MCF7‐derived tumors administered with PBS (−Met) or metformin (+Met) in (B). (E) is the quantification of (D). F) Representative IHC (immunohistochemistry) staining images of PRMT6 and H3R2me2a in xenograft tumors administered with PBS (−Met) or metformin (+Met) in (B). G) RT‐qPCR analysis of the transcription of *FEN1*, *POLA2*, and *POLD1* in WT and PRMT6‐E164A MCF7‐derived tumors administered with PBS (−Met) or metformin (+Met) in (B). H–J) Metformin inhibited the xenograft growth of control (Ctrl) but not PRMT6‐overexpressing (PRMT6 OE) MCF7 cells. Xenograft mice were administrated with PBS (−Met) or metformin (+Met) (10 mice group^−1^). Quantification of the average volume of tumors over time was shown in (H). The dissected tumors and quantification of the tumor weight were shown in (I,J). K,L) Immunoblots of PRMT6 and H3R2me2a in control (Ctrl) and PRMT6‐overexpressing (PRMT6 OE) MCF7‐derived tumors administered with PBS (−Met) or metformin (+Met) in (L). The relative intensity of PRMT6/Actin and H3R2me2a/H3 was set at 1 in control (‐Met). M) Representative IHC staining images of PRMT6 and H3R2me2a in xenograft tumors administered with PBS (−Met) or metformin (+Met) in (I). For (A–C,H–J), data represent means ± SEM; *n* = 10 biological independent experiments. For (E,G), and l, data represent means ± SEM; *n* = 3 biological independent experiments. Two‐tailed *t*‐tests (C,E,G,J,L) and two‐tailed paired *t*‐tests (A,H) were used for statistical analysis. * *P *< 0.05, ** *P *< 0.01, *** *P *< 0.001, *n.s*., no significance.

We also performed xenograft experiments to examine the effect of metformin on the growth of WT and PRMT6‐E164A MCF7 cells. Metformin significantly impaired the growth of xenograft tumors derived from WT but not PRMT6‐E164A mutant (**Figure** **6**A–C). Metformin also reduced PRMT6 and H3R2me2a in WT but not in PRMT6‐E164A tumors (Figure 6D–F). RT‐qPCR analysis revealed that the transcription of *FEN1*, *POLA2*, and *POLD1* was repressed by metformin in WT but not in PRMT6‐E164A tumors (Figure 6G).

**Figure 7:**
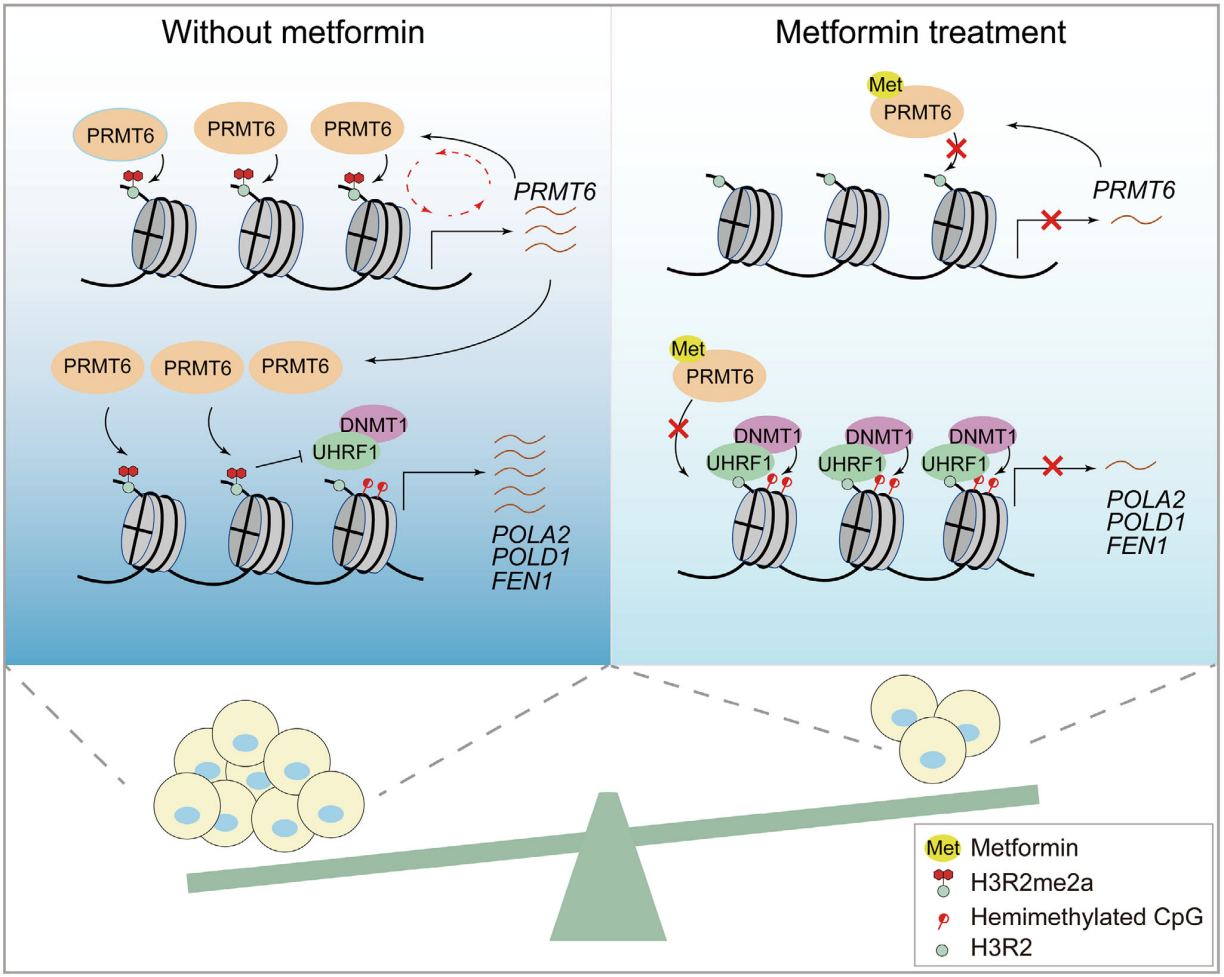
Proposed model for metformin impairs breast cancer growth by regulating PRMT6‐H3R2me2a‐5mC axis. In breast cancer cells, PRMT6 catalyzes H3R2me2a at the promoter regions of DNA replication‐related genes, which prevents DNA methylation by DNMT1 and facilitates their transcription. When cells are treated with metformin, metformin directly binds PRMT6 and inhibits it activity to catalyze H3R2me2a, which in turn represses *PRMT6* transcription and further reduces the occupancy of H3R2me2a at the promoter regions of DNA replication‐related genes. UHRF1 then binds and recruits DNMT1 to catalyze 5mC at the promoter regions of these genes, leading to their transcription repression and growth inhibition.

We next investigated whether *PRMT6* overexpression can attenuate the detrimental effect of metformin. Metformin significantly impaired the growth of xenograft tumors derived from control but not *PRMT6*‐overexpressing MCF7 cells (Figure 6H–J). Both immunoblots and immunohistochemistry revealed that metformin reduced PRMT6 and H3R2me2a in control, and this effect was reverted by *PRMT6* overexpression (Figure 6K‐M). Collectively, these data indicate that metformin impairs tumor growth primarily by inhibiting PRMT6‐catalyzed H3R2me2a, which then increased DNA methylation and repressed the transcription of DNA replication‐associated genes (**Figure** **7**).

## 3 Discussion

Metformin is the world's widely prescribed oral medication for the treatment of type 2 diabetes. Although metformin inhibits tumor growth, it remains to be determined about metformin's anti‐cancer properties and the direct molecular mechanism. Here, we report that metformin impairs breast cancer growth primarily by targeting histone methyltransferase PRMT6. Mechanistically, metformin directly binds PRMT6 and inhibits its activity to catalyze H3R2me2a, which in turn represses *PRMT6* transcription and further decreases H3R2me2a. By reducing PRMT6‐catalyzed H3R2me2a, metformin enhances the association of UHRF1 with chromatin to promote DNA hypermethylation and repress the expression of DNA replication‐related genes, leading to retarded DNA replication and cell cycle arrest (Figure 7). We also identified the E164 residue of PRMT6 is critical for its binding to metformin. Mutation of PRMT6‐E164A alleviates the inhibitory effect of metformin on breast cancer cell growth and tumorigenesis. Moreover, metformin in combination with a DNA replication inhibitor synergistically inhibits tumor growth. Our results thus uncover a novel anti‐cancer target of metformin in breast cancer cells.

Metformin has been reported to inhibit the growth of cancer cells by activating AMPK, including hepatoma cells, colon, prostate cancer cells, etc.^[^ ^10^, ^31^, ^40^, ^41^, ^42^ ^]^ However, this effect appears to be tissue and cancer‐type specific. For example, metformin has been shown to inhibit mTOR, regulate glycolysis and the TCA cycle, and repress cell growth independent on AMPK in MEFs, prostate cancer LNCaP cells, breast cancer MCF7, and lung cancer A549 cells.^[^ ^15^, ^16^, ^17^ ^]^ Recent work has shown that AMPK attenuates rather than boosts the anti‐cancer effect of metformin by accelerating glycolysis.^[^ ^18^ ^]^ Here, we identify the histone arginine methyltransferase PRMT6 as a new anti‐cancer target for metformin using multiple techniques, including DARTS, mass spec, and ITC. Although metformin may inhibit cancer growth through complex mechanisms, the inhibition of PRMT6 activity and subsequent repression of PRMT6 expression primarily explain the anti‐tumor activity of metformin in breast cancer. Interestingly, we noted that there is an anti‐correlation between the expression of PRMT6 and AMPK. The expression of AMPK in HepG2 is higher than that in MCF7, MDA‐MB‐436, and MDA‐MB‐468, while the expression of PRMT6 in HepG2 is much lower. This may explain why AMPK is required for metformin to repress the growth of HepG2, and PRMT6 is required for metformin to inhibit the growth of breast cancer cells (MCF7, MDA‐MB‐436, MDA‐MB‐468). In addition, the expression of PRMT6 in MCF7, MDA‐MB‐436, and MDA‐MB‐468 is higher than that in MDA‐MB‐231, which correlates with their sensitivity to metformin. Moreover, we found that AMPK represses PRMT6 expression through knockdown and overexpression of *AMPK*. This relationship between AMPK and PRMT6 can be used to develop combination therapies to treat cancer. In fact, the combined use of metformin and compound C, the AMPK inhibitor, synergistically inhibits the growth of MDA‐MB‐231, which is less sensitive to metformin treatment alone. The reason could be that inhibition of AMPK with compound C elevates PRMT6 expression to make cells sensitive to metformin. Further investigation indicated that AMPK promotes the reduction of PRMT6 protein stability. It is highly plausible that AMPK may mediate this effect through phosphorylation of PRMT6, thereby facilitating its degradation. Future studies are warranted to validate this phosphorylation‐dependent mechanism.

PRMT6 is overexpressed in multiple types of cancer, including breast cancer, prostate cancer, bladder tumor, gastric cancer, and lung cancer, and its high expression is associated with poor overall survival.^[^ ^26^, ^27^, ^28^, ^29^, ^30^, ^43^ ^]^ PRMT6 has been thought to act as an oncogene that represses the functions of p53, p21, and p16 to regulate cell cycle and apoptosis.^[^ ^44^, ^45^ ^]^ In breast cancer, the expression of PRMT6 positively correlates with tumor stages, suggesting that PRMT6 may contribute to tumor progression.^[^ ^46^ ^]^ *PRMT6* overexpression contributes to DNA hypomethylation, which could lead to genomic instability and expression of cancer‐promoting genes.^[^ ^20^ ^]^ Here, we found that PRMT6 catalyzes H3R2me2a to promote itself transcription, which forms a feed‐forward loop. By inhibiting PRMT6 activity and repressing PRMT6 expression, metformin augments DNA methylation at the promoter regions of DNA replication‐related genes, leading to their transcriptional repression. In addition, PRMT6 has been reported to methylate non‐histone proteins, including regulator of chromatin condensation 1 (RCC1), CRAF, etc.^[^ ^47^, ^48^ ^]^ Although we cannot rule out the possibility that PRMT6 may methylate other proteins in breast cancer cells, our results with the H3R2A mutant suggest that metformin primarily inhibits PRMT6‐catalyzed H3R2me2a to exert anti‐cancer activity.

Although inhibition of PRMT6 could theoretically lead to functional compensation by other PRMT family members, our data provide multiple lines of evidence against this possibility. First, RNA‐Seq and RT‑qPCR analysis showed no upregulation of PRMT family genes following metformin treatment (Figure 3C; Figure S5C, Supporting Information). Second, enzymatic profiling revealed that metformin has minimal inhibitory activity against other histone methyltransferases in the PRMT family, including PRMT1, PRMT4, PRMT5, and PRMT7 (Figure S3C,D, Supporting Information). Finally, and most critically, functional rescue experiments demonstrated that only the overexpression of PRMT6 but not of any other PRMT member (PRMT1, PRMT2, PRMT3, PRMT5, PRMT7, and PRMT9) was able to attenuate the growth‐inhibitory effects of metformin (Figure S6P–V, Supporting Information). Collectively, these results argue that compensatory mechanisms within the PRMT family are unlikely to occur upon PRMT6 inhibition.

As PRMT6 is considered as a potential anti‐cancer therapy target, several PRMT6 inhibitors have been developed. These PRMT6 inhibitors include MS049, EPZ020411, licochalcone A, etc., which have been shown to inhibit the growth of breast cancer, colorectal cancer, and melanoma.^[^ ^26^ ^]^ Compared with these inhibitors, metformin could be a low‐cost and less side‐effect candidate for anti‐cancer therapy for breast cancer. Notably, our work revealed that metformin synergistically inhibits cancer cell growth when combined with a DNA replication inhibitor (HU), suggesting that metformin can be used in combination with other therapies.

Our tumor xenograft models were established according to the well‐established protocols,^[^ ^49^ ^]^ which did not account for potential effects mediated by estrogen signaling. We analyzed the RNA‐seq dataset for estradiol‐treated mouse breast tissue (GSE130032) and MCF7 cells (GSE56066). It revealed no significant alterations in *PRMT6* transcription following estrogen exposure, suggesting that estrogen may not directly modulate the interaction between metformin and PRMT6. Nonetheless, the estrogen signaling merits deeper investigation in our future research.

Aberrant DNA methylation patterns, including global hypomethylation and regional hypermethylation, are associated with cancer and implicated in oncogenic events.^[^ ^20^ ^]^ Metformin has been reported to induce DNA hypermethylation by interfering with cell metabolism.^[^ ^21^ ^]^ Metformin decreases the synthesis of SAH, a strong feedback inhibitor of DNA methyltransferases, while promotes the accumulation of SAM, the universal methyl donor.^[^ ^21^ ^]^ PRMT6 negatively regulates DNA methylation, and its up‐regulation contributes to DNA hypomethylation in breast cancer.^[^ ^20^ ^]^ Metformin increased SAM in both WT and PRMT6‐E164A MCF7 cells, and no difference was observed between WT and PRMT6‐E164A (Figure S7A, Supporting Information). As metformin has no effect on DNA methylation in PRMT6‐E164A and H3R2A mutants (Figure 4A,B; Figure S8E,F, Supporting Information), metformin induces DNA hypermethylation in MCF7 cells not by increasing SAM accumulation but primarily by inhibiting PRMT6‐catalyzed H3R2me2a.

Metformin has been reported to reduce histone H3K27me3 and H3K4me3 in prostate organoids and *Caenorhabditis elegans*.^[^ ^22^, ^23^ ^]^ The inhibitory effect of metformin on H3K27me3 and H3K4me3 is related to its functions in activating AMPK. As AMPK is dispensable for metformin to reduce the proliferation of breast cancer cells (MCF7, MDA‐MB‐436, MDA‐MB‐468), it is not surprising that metformin has no effect on these histone markers. Instead, we observed the specific reduction of PRMT6‐catalyzed H3R2me2a in metformin‐treated breast cancer cells. To our knowledge, this is the first time to report that metformin inhibits PRMT6‐catalyzed histone H3R2 methylation. As the expression of AMPK and PRMT6 varies dramatically in cell lines studied (Figure 1G), the heterogeneity in histone modification responses to metformin may reflect the intrinsic differences in the cancer subtypes from which the cell lines were derived. Another reason may be related to the high concentration of metformin (50 mm) used to reduce H3K4me3 in *Caenorhabditis elegans*.^[^ ^23^ ^]^

Given the scarcity of clinical data from metformin‐treated tumor patients, we turned to whole blood transcriptome data of type 2 diabetes patients receiving metformin administration. Our analysis revealed that metformin downregulates *PRMT6* transcription in peripheral blood (Figure S9C, Supporting Information). In addition, analysis of the breast cancer patient dataset GSE169246 showed a positive correlation of PRMT6 expression between tumor tissues and peripheral blood (Figure S12, Supporting Information). These findings suggest that metformin may lower PRMT6 in the peripheral blood and tumor tissues of cancer patients, and position PRMT6 as a promising biomarker for metformin‐based breast cancer therapy. Nevertheless, we acknowledged that a limitation of this study is the absence of direct clinical evidence linking metformin treatment to PRMT6 expression in human tumors. To address this specific point, future clinical trials specifically designed to measure PRMT6 expression in paired tumor and blood samples from patients undergoing metformin treatment will be essential.

In summary, we demonstrate that metformin impairs breast cancer growth primarily via a PRMT6‐dependent mechanism. Metformin directly binds PRMT6 and inhibits its activity to catalyze H3R2me2a, which in turn represses *PRMT6* transcription and further reduces H3R2me2a. As a consequence of reduced H3R2me2a, metformin promotes DNA hypermethylation to repress the transcription of DNA replication‐related genes and impair tumorigenesis. Our work thus identifies a novel anti‐cancer target of metformin in breast cancer cells.

## 4 Experimental Section

### Reagents or Resources

A comprehensive overview of the reagents and resources utilized in this study is provided in Tables S1–S3 (Supporting Information).

### Data and Code Availability

All data supporting the findings of this study are included in the manuscript and its Supporting Information. The accession numbers for ChIP‐seq of H3R2me2a are PRJNA810196. The accession number for RNA‐seq of metformin‐treated WT and PRMT6‐E164A mutant is PRJNA858970. The whole blood transcriptome data for type 2 diabetes patients administrated with metformin for three months were retrieved from GSE153792.

### Animals

All procedures were approved by the Animal Care and Use Committee of Hubei University. MMTV‐PyMT transgenic mice were purchased from Nanjing Model Animal Center. All mice were born and raised under pathogen‐free conditions at 20–24 °C, 40–70% humidity, and a 12/12‐h light‐dark cycle. Water and food were provided by the Animal Center of the School of Life Sciences, Wuhan University. For the metformin treatment assay, the MMTV‐PyMT female mice were divided into two groups (10 mice group^−1^). From 40 days, one group was fed with PBS, and the other group was fed with 60 mg kg^−1^ metformin every two days. The size and number of mammary tumors were measured and recorded every four days. At the third month, the mice were sacrificed and dissected. The xenograft mouse studies were established according to the well‐established protocols.^[^ ^49^ ^]^ The BALB/c nude mice were purchased from Beijing Vital River Laboratory Animal Technology Co., Ltd. (Beijing, China) and handled in accordance with the guidance for the local care and use of laboratory animals under specific pathogen‐free conditions. The mice were subcutaneously injected with 2 × 10^6^ MCF7 or MDA‐MB‐468 cells and 0.1 mL matrix gel. When the tumor diameter reached 4–5 mm, the mice were divided into two groups (5–10 mice group^−1^). One group was fed with 60 mg kg^−1^ metformin every two days for four weeks, while the other group was given PBS every two days. This dosage of metformin was determined in preliminary experiments that manifest tumoricidal action without any apparent cytotoxicity on normal mice. The mice were humanely sacrificed, and the weight of the resulting tumors was measured according to the approved guidelines.

### Cell Lines and Cell Culture

The MCF7, MDA‐MB‐231, T47D, HeLa, and HepG2 cells were obtained from American Type Culture Collection (ATCC). The MDA‐MB‐436 and MDA‐MB‐468 were purchased from Procell Life Science Technology (Wuhan, China). MCF7 cells were grown in minimum essential medium (MEM) supplemented with 10% FBS, 1% penicillin/streptomycin, and 3 mg mL^−1^ insulin. The other cell lines were grown in Dulbecco's modified Eagle's medium (DMEM) supplemented with 10% fetal bovine serum and 1% penicillin/streptomycin. All cell lines used in this study were reauthenticated by short tandem repeat analysis after resuscitation in the laboratory. WT and PRMT6‐E164A MCF7 cells were constructed by CRISPR‐Cas9. In brief, p459‐gPRMT6 and repair plasmid (WT PRMT6, PRMT6‐E164A) were co‐transfected into MCF7 cells. After 48 h, cells were selected with 2 µg mL^−1^ puromycin. The single clones were then picked and expanded into 96‐well plates. The knock‐in efficiency was verified by DNA sequencing. The sequence of gPRMT6 used is: TGGATGCCATCGTGAGCGAG.

### Plasmids and Transfection

The expression plasmids were constructed by standard molecular biology techniques. A point mutation was generated by site‐directed mutagenesis. Breast cancer cells (MCF7, MDA‐MB‐436, MDA‐MB‐468) were infected with lentiviruses to stably overexpress genes of interest.^[^ ^33^ ^]^ To knock down gene expression, cells were transfected with 5 µL siRNA with reagent siRNA Mate (G04003, GenePharma).^[^ ^33^ ^]^ To construct stable knockdown cell lines, shRNA hairpins were cloned into the lentiviral vector pLKO.1.^[^ ^33^ ^]^ A control hairpin that targeted GFP was cloned into the pLKO.1 vector and used as a negative control. HEK293T cells were transfected with pLKO.1 vectors and lentiviral packaging vectors. Forty‐eight hours later, supernatant‐containing lentivirus was collected. Breast cancer cells were transfected with lentivirus, and selection was performed under 2 µg mL^−1^ puromycin. The knockdown efficiency was examined by immunoblots and/or quantitative reverse transcription PCR (RT‐qPCR). AMPK silencing was achieved by dual knockdown of both AMPK*α1* and *AMPKα2* as described.^[^ ^50^ ^]^

### Cell Counting, CCK‐8, and Colony Formation Assay

For the cell counting assay, cells were seeded into 12‐well plates at 2–5 × 10^4^ cells well^−1^ and treated with metformin. Cells were collected at different time points and counted using the cell counter (HSCORE, China). For the CCK‐8 assay, cells were seeded into 96‐well plates at 2 × 10^3^ cells well^−1^ and treated with metformin. After 48 h, cell proliferation rate was determined by the Cell Counting Kit (CCK‐8, Dojindo, Japan) according to the manufacturer's instructions. Normalization was done to cells treated with the same concentration of NaCl. For colony formation assay, cells were seeded into 12‐well plates at 2 × 10^3^ cells well^−1^, treated with metformin, and then cultured for 14 days.

### Quantitative Reverse Transcription PCR (RT‐qPCR)

Total RNA was extracted from cells with TRIzol reagent RNAiso Plus (Takara). The purified RNA was treated with DNase (Sigma) to remove contaminated DNA and reversed transcribed into cDNA using a reverse transcriptase kit (M‐MLV) (Abclonal). The cDNA was quantitated by qPCR with SYBR Green premix (Abclonal) using primers listed in Table S2 (Supporting Information). The mRNA level of the gene of interest was normalized to that of *ACTIN*.

### Immunoblot Analysis

Cells were lysed with RIPA lysis buffer (Biosharp) containing 1 mm PMSF for 5 min, sonicated for 1 min and boiled with SDS loading buffer. Frozen tissue was homogenized in tissue lysis buffer (20 mm Tris‐HCl pH7.5, 150 mm NaCl, 1 mm EDTA, 1% sodium deoxycholate, 1% Triton X‐100) supplemented with protease inhibitor cocktails and boiled with SDS loading buffer. The supernatant was subjected to SDS‐PAGE and transferred to a 0.2 µm polyvinylidene fluoride membrane (PVDF, Bio‐Rad). The membrane was blocked with 5% skim milk or BSA, incubated with indicated primary antibodies followed by horseradish peroxidase (HRP)‐labelled IgG secondary antibodies, and developed with ECL chemiluminescence detection kit (Biosharp) on a ChemiDoc imaging system (Bio‐Rad, USA). The band intensity was quantified with Image J software.

### Drug Affinity Responsive Target Stability (DARTS)

DARTS was performed as described.^[^ ^33^ ^]^ Cells were lysed in M‐PER buffer (Thermo Scientific, 78 501) with protease inhibitors (MCE, HY‐K0010). TNC buffer (50 mm Tris‐HCl pH8.0, 50 mm NaCl, 10 mm CaCl_2_) was then added to the cell lysate, and protein concentration was determined by the BCA protein assay kit (CWBIO, CW0014S). The lysate was incubated with 0–0.5 mm metformin for 1 h on ice followed by 20 min incubation at room temperature. Digestion was performed using 50 µg mL^−1^ pronase (Roche, 10 165 921 001) at room temperature for 0.5 h and quenched by adding excess protease inhibitors. The lysate was subjected for mass spectrometry or immunoblots as described.^[^ ^33^ ^]^

### Isothermal Titration Calorimetry (ITC) Assay

ITC assay was performed using the Nano ITC (TA Instruments) at 25 °C as described.^[^ ^51^ ^]^ For all experiments, the initial injection of 0.5 µL metformin solution was discarded to eliminate the effect of titrant diffusion across the syringe tip during the equilibration process, and each dataset consisted of 20 injections of 2.5 µL each of 80 µm metformin into a sample cell containing 500 µL of 4 µm purified PRMT6. The dissociation constant (Kd) and other thermodynamic parameters were determined by fitting the integrated titration data using the independent model implemented in Nanoanalyzer software (v.3.7.5).

### RNA Sequencing (RNA‐seq)

WT and PRMT6‐E164A MCF7 cells were treated with 1 mm metformin for 48 h. Total RNA was then extracted using TRIzol (Invitrogen) as described.^[^ ^52^ ^]^ Library construction, sequencing, and bioinformatics analysis were done by Origingene Bio‐pharm Technology Co. Ltd. (Shanghai). The differentially expressed genes (DEGs) were defined as log_2_(FC) ≥ 1 or log_2_(FC) ≤ −1, *P *≤ 0.05. DEGs were used for KEGG pathway analysis, and KOBAS software was used to test the statistical enrichment of DEGs in KEGG pathways.

### Histone Methyltransferase (HMT) Assay

Purified PRMT6 (0.4 µg) was incubated with 0.2 µg purified recombinant histone H3, 0.5 µg in vitro assembled octamers or nucleosomes in 20 µL HMT buffer (100 mm Tris‐HCl pH8.6, 0.04% TritonX‐100, 4 mm MgCl_2_, 2 mm Tris (2‐carboxyethyl)phosphine hydrochloride, 31.3 µm SAM, protease inhibitor cocktail) at 37 °C. The reaction products were then subjected to immunoblots with anti‐H3R2me2a and anti‐H3 antibodies.

### Immunofluorescence

Cells were fixed with 4% paraformaldehyde and permeabilized with 0.2% Triton X‐100 in PBS at room temperature for 15 min. Cells were then incubated with primary antibody at 4 °C overnight followed by incubating with Alexa Fluor‐conjugated secondary antibody (Invitrogen) at 30 °C for 1 h. Cells were washed with cold PBS, subjected to 1 µg mL^−1^ 4,6‐diamidino‐2‐phenylindole (DAPI) staining for 10 min, and visualized by ZEISS LSM710 microscope using ZEN Imaging Software (ZEISS).

### Pulldown Assay

Five micro grams of recombinant His‐PRMT6 or His‐PRMT6‐E164A was incubated with 0.5 µg nucleosomes in the presence or absence of 0.5 mm metformin in 600 µL binding buffer (50 mm Tris‐HCl pH7.5, 150 mm NaCl, 0.05% NP‐40, protease inhibitor cocktail). His‐PRMT6 was immunoprecipitated with Nickle beads for 4 h at 4 °C. The beads were then washed with 1 mL washing buffer (50 mm Tris‐HCl pH7.5, 300 mm NaCl, 0.05% NP‐40, protease inhibitor cocktail) for three time followed by boiling in 60 µL 2 × SDS loading buffer for 5 min. The supernatant was used for immunoblots.

### Molecular Dynamics Simulations

The molecular docking was performed as described.^[^ ^51^ ^]^ The crystal structure of PRMT6 was obtained from the Protein Data Bank (PDB: 4QQK). To visualize the docked conformation, the PyMol molecular graphics system was used, which removed water molecules. The molecular docking simulation of PRMT6 and metformin was performed with AutoDock Vina.

### Methylated DNA Immunoprecipitation (MeDIP)

Genomic DNA was extracted from cells in buffer A (10 mm Na_3_PO_4_ pH7.0, 1.4 m NaCl, 0.5% Triton X‐100). The DNA was sheared by sonication, subjected to immunoprecipitation with anti‐5mC antibody at 4 °C for 6 h followed by incubation with Protein G Dynabeads (Invitrogen) at 4 °C for 2 h. The beads were washed and the eluted DNA/protein complexes were treated with 20 µg proteinase K at 55 °C for 2 h and digested at 65 °C overnight. The purified DNA was then treated with RNase A to remove RNA and quantitated by qPCR.

### Subcellular Fractionation

Cells were rinsed with ice‐cold PBS and resuspended in buffer B (10 mm HEPES pH7.9, 100 mm KCl, 1 mm EDTA pH8.0, 0.1 mm EGTA pH8.0, 1 mm DTT, 0.15% NP‐40, 1 mm PMSF, protease inhibitor cocktail) for 20 min on ice. After centrifugation at 12 000 g for 10 min, the supernatant was collected as the cytoplasmic fraction. The pellet was washed with cold buffer B and then resuspended in buffer C (3 mm EDTA pH8.0, 0.2 mm EGTA, 1 mm DTT, 1 mm PMSF, protease inhibitor cocktail). After centrifugation, the supernatant was collected as the nuclear matrix. The pellet was washed with cold buffer C and then resuspended with 2× loading buffer as the chromatin fraction.

### Bisulfite Sequencing

The bisulfite sequencing was performed as described.^[^ ^20^ ^]^ Bisulfite conversion was performed using the DNA Methylation Kit (CWBIO, CW2140M). The PCR products were cloned using Takara Premix (Takara, R040A), and individual clones were sequenced.

### Chromatin Immunoprecipitation (ChIP)

Cells were cross‐linked with 1% formaldehyde and quenched by 0.125 m glycine. Cells were collected, washed, and lysed in lysis buffer B (50 mm Tris pH8.0, 5 mm EDTA, 1% SDS, 1 mm PMSF, protease inhibitor cocktail). DNA was sheared by sonication and subjected to immunoprecipitation with antibodies pre‐bound to Protein G Dynabeads (Invitrogen) overnight. The beads were washed, and the eluted DNA/protein complexes were treated with 20 µg proteinase K at 55 °C for 2 h and reversed at 65 °C overnight. The purified DNA was treated with RNase A and quantitated by qPCR. For ChIP‐seq, library construction and sequencing were performed by SEQHEALTH Technology Co. Ltd. (Wuhan, China) and analyzed as descried.^[^ ^51^ ^]^

### Dual‐Luciferase Reporter Assay

The pGL3‐PRMT6 plasmid was constructed by inserting the promoter sequence of *PRMT6* into the pGL3‐basic vector (Promega). For the dual‐luciferase reporter assay, cells were cultured to 70–80% confluence in a 24‐well plate. 200 ng pGL3‐PRMT6 in combination with 40 ng pRT‐TK were transfected into cells, followed by metformin treatment for 48 h. Cells were then collected and detected with a dual‐luciferase report assay system (Promega, E1910).

### Cell Cycle Analysis

For cell cycle analysis, cells were treated with or without 1 mm metformin for 48 h. Cells were collected and fixed with 70% ethanol overnight. Cells were then stained with 50 µg mL^−1^ propidium iodide (PI) and measured by Flow cytometry (CytoFLEX, USA) as described previously.^[^ ^52^ ^]^ The data were analyzed with Modfit LT 4.1 according to the manufacturer's instructions.

### RNA Stability

MCF7 cells were treated with the transcriptional inhibitor Actinomycin D (20 µg mL^−1^; MCE). Total RNA was extracted at various time points (0, 4, 6, and 8 h) following treatment. The relative abundance of target mRNAs was determined by RT‐qPCR and normalized to *ACTIN*. The mRNA half‐life was subsequently calculated based on the decay rate over time.

### Whole‐Genome Bisulfite Sequencing

MCF7 cells were treated with 1 mm metformin or left untreated as controls. Genomic DNA was extracted from the collected cells using a standard phenol‐chloroform method. Whole‐genome bisulfite sequencing was performed by Berry Genomics (Wuhan, China) on an Illumina platform. Briefly, DNA was fragmented, converted using bisulfite treatment, and sequenced to generate paired‐end reads. Bioinformatic analysis was conducted using the Bismark software for alignment and methylation calling. Differentially methylated regions (DMRs) were identified with a threshold of adjusted *P*‐value < 0.05 and methylation difference > 2. Functional enrichment analysis of DMR‐related genes was performed using KEGG pathways.

## Conflict of Interest

The authors declare no conflict of interest.

## Author Contributions

Y.W. and X.X. contributed equally to this work. G.C., X.Y., and S.L. designed research. Y.W., X.X., X.Y., and M.W. performed research. Y.W., X.X., Y.T., F.G., M.W., and Y.W. analyzed data. X.Y. and S.L. wrote the paper with input from all authors.

## Supporting information



Supporting Information

## Data Availability

The data that support the findings of this study are available from the corresponding author upon reasonable request.;
