# Supplementary material for: Metformin Impairs Breast Cancer Growth through the Inhibition of PRMT6
Source: Adv Sci (Weinh). 2025 Dec 1;13(8):e08525. doi: 10.1002/advs.202508525 (PMC12884805; doi:10.1002/advs.202508525)
Supplement: Supplementary file 1 — Supporting Information [file ADVS-13-e08525-s001.pdf]

Metformin impairs breast cancer growth through the inhibition of PRMT6

Yinsheng Wu[1,5], Xinlin Xu[1,5], Yue Tong[1], Min Wang[2], Feng Ge[2], Min Wu[3], Yunlong Wang[1], Gang Chen[4, *], Xilan Yu[1, *], Shanshan Li[1, *]

[1]School of Life Sciences, Hubei University, Wuhan, Hubei 430062, China

[2]Key Laboratory of Algal Biology, Institute of Hydrobiology, Chinese Academy of Sciences, Wuhan, Hubei 430072, China

[3]College of Life Sciences, Wuhan University, Wuhan, 430072, Hubei, China

[4]Hubei University of Chinese Medicine, Wuhan 430065, PR China; Department of Geriatrics, Hubei Provincial Hospital of Traditional Chinese Medicine, Affiliated Hospital of Hubei University of Chinese Medicine, Wuhan 430061, PR China; Hubei Shizhen Laboratory, Wuhan 430065, PR China

[5]These authors contribute equally to this work

*Corresponding authors: Shanshan Li, shl@hubu.edu.cn

Xilan Yu, yuxilan@hubu.edu.cn

Gang Chen, chengang@hbucm.edu.cn

Supplemental Data

1. Supplementary Figure Legends.

2. Supplementary Figure (Figures S1-S12).

3. Supplementary Table (Table 1-3).

SUPPLEMENTARY FIGURE LEGENDS

Figure S1. Metformin impairs the growth of breast cancer cells.

(A) Effect of metformin on growth of cell lines (MDA-MB-468, MCF7, MDA-MB-436, T47D). Cells were treated with 0-0.5 mM metformin as indicated. (B) Effect of metformin on growth of cell lines (MDA-MB-468, MCF7, MDA-MB-436, T47D, MDA-MB-231, HepG2, HeLa). Cells were treated with 0-5 mM metformin as indicated. (C) Metformin inhibited the xenograft growth of MCF7 cells. Xenograft mice were administrated with PBS (- Met) or 60 mg/kg metformin (+ Met) by oral gavage (5 mice/group). The xenograft tumors were measured over time and dissected at the endpoint. The dissected tumors and quantification of the tumor weight was shown. (D) Metformin inhibited tumor growth in spontaneous breast cancer mouse model (MMTV transgenic mice). The MMTV-PyVT 634Mul/J mice were treated with PBS (-Met) or 60 mg/kg metformin (+Met) by oral gavage (14 mice/group). The tumors were dissected at the endpoint. The dissected tumors and quantification of the tumor weight was shown. (E) Effect of metformin (Met) on growth of control (shCtrl) and *AMPK*-knockdown (shAMPK) MDA-MB-468 cells. Cells were treated with 1 mM metformin. (F) Effect of metformin (Met) on growth of control (shCtrl) and *PEN2*-knockdown (shPEN2) MCF7 cells. Cells were treated with 1 mM metformin. *PEN2* knockdown efficiency was determined by immunoblots. (G) Immunoblot analysis of AMPK expression in the lysates of T47D, MDA-MB-231, MCF7, HeLa and HepG2 cells.

For A, B, E-F, data represent means ± SEM; n=3 biological independent experiments. For C, data represent means ± SE; n=5 biological independent experiments. For D, data represent means ± SEM; n=14 biological independent experiments. Two-tailed *t*-tests (C, D) and two-tailed paired

t-tests (A, B, E, F) were used for statistical analysis. *, $P<0.05$; **, $P<0.01$; ***, $P<0.001$. For G, shown are the typical example of three biological independent experiments.

Figure S2. Metformin inhibits the growth of breast cancer cells by binding to PRMT6.

(A) Metformin binds to PRMT6 as determined by DARTS. MDA-MB-436 and MDA-MB-468 cell lysates were incubated with 0-0.5 mM metformin (Met) followed by pronase digestion. The digested products were resolved on SDS-PAGE followed by immunoblots. Actin was served as an endogenous control. (B) Metformin binds to PRMT6 as determined by microscale thermophoresis (MST) assay. (C) CCK-8 cell proliferation assay showing knockdown of PRMT6 attenuated the inhibitory effect of metformin on MCF7 cell growth. (D) Effect of 1 mM metformin (Met) on growth of control (shCtrl) and *PRMT6*-knockdown (shPRMT6) MDA-MB-436 and MDA-MB-468 cells. (E and F) Effect of 0.5 or 5 mM metformin (Met) on growth of shCtrl and shPRMT6 MDA-MB-231 cells. (G) Effect of 1 mM metformin (Met) on growth of control (shCtrl), *EZR*-knockdown (shEZR), *DDX39A*-knockdown (shDDX39A), *DDX39B*-knockdown (shDDX39B), *PABPC1*-knockdown (shPABPC1) and *OTUB1*-knockdown (shOTUB1) MCF7 cells. The shEZR and shOTUB1 groups share the same data of shCtrl and shCtrl+Met. (H) Effect of 1 mM metformin (Met) on growth of control (shCtrl) and *PRMT6*-knockdown (shPRMT6) HepG2 cells. (I) TCGA analysis of the transcription of *AMPK* (*PRKAA1*, *PRKAG1*) and *PRMT6* in different cancer cell lines. Shown are $\log_2$(*PRKAA1/PRMT6*) and $\log_2$(*PRKAG1/PRMT6*). (J) Analysis of the transcription of *PRMT6* and *PRKAA1* in normal and primary breast tumor tissues. Two-tailed *t*-test was used for statistical analysis. (K) Immunoblot analysis of the effect of *AMPK* knockdown (shAMPK) or *AMPK* overexpression (AMPK OE)

on the levels of PRMT6 in MCF7, MDA-MB-436, MDA-MB-468 and HepG2 cells. (L) ChIP-qPCR analysis of AMPK occupancy at *PRMT6* promoter regions in MCF7 cells. H1 served as a positive control. (M) RT-qPCR analysis of *PRMT6* mRNA stability in shCtrl and shAMPK MCF7 cells treated with 20 μg/ml actinomycin D for 0-8 h. (N) Immunoblot showing PRMT6 levels in shCtrl and shAMPK MCF7 cells treated with 100 μg/ml cycloheximide for 0-8 h. (O) Effect of metformin and compound C on growth of MDA-MB-231 cells. Cells with treated with 1 mM metformin with or without 2 μM compound C. (P) Effect of 1 mM metformin (Met) on growth of shCtrl and shAMPK MDA-MB-231 cells.

For C, D, F-H, L, M, O, P, data represent means ± SEM; n=3 biological independent experiments, two-tailed paired *t*-tests were used for statistical analysis. *, $P<0.05$; **, $P<0.01$; ***, $P<0.001$. For A, K and N, shown are the typical example of at least biological independent experiments.

Figure S3. Metformin directly inhibits PRMT6-catalyzed histone H3R2me2a.

(A and B) In vitro HMT assay showing metformin inhibits the activity of PRMT6 to catalyze H3R2me2a in a dose-dependent manner. The recombinant PRMT6 was purified from *E. coli*. The in vitro HMT assay was performed with 0.4 μg purified PRMT6 and 0.5 μg in vitro assembled octamers (A) or nucleosomes (B) at 37°C for 0.5 h. (C and D) In vitro HMT assay showing metformin inhibited the activity of PRMT6 but not PRMT1, PRMT4, PRMT5 and PRMT7. The PRMT6, PRMT1, PRMT4, PRMT5 and PRMT7 were purified from 293T cells. (E) DARTS analysis of the interaction between metformin and PRMT6 truncation mutants. (F) Left panel: Molecular docking assay of metformin binding to PRMT6-SAMD domain. Right panel: DARTS experiments showing metformin bound PRMT6-SAMD but not PRMT6-$D_{150-170}$

truncation mutant. (G) Sequence alignment of PRMT6 in a wide range of organisms. (H) DARTS experiments showing mutation of PRMT6-Y51F and PRMT6-S165P abolished the interaction between metformin and PRMT6. The purified WT PRMT6, PRMT6-Y51F, PRMT6-L162M and PRMT6-S165P were incubated with 0-0.5 mM metformin (Met) followed by digestion with pronase. The digested products were analyzed by immunoblots. Actin was served as an endogenous control. (I) Metformin directly binds to WT PRMT6 but not PRMT6-E164A or PRMT6-E155A as determined by ITC experiments. (I) In vitro HMT assay showing metformin repressed the activity of WT PRMT6 but not PRMT6-Y51F and PRMT6-E155Q. (K) In vitro HMT assay showing metformin reduced the activity of PRMT6-L162M but had no effect on the activity of PRMT6-S165P. (L) DNA sequencing results validated the PRMT6-E164A MCF7 cell line. (M) H3R2me2a staining of WT and PRMT6-E164A MCF7 cells treated with or without 1 mM metformin. (N) CCK-8 cell proliferation assay showing mutation of PRMT6-E164A attenuated the inhibitory effect of metformin on cell growth. WT and PRMT6-E164A MCF7 cells were grown in MEM medium supplemented with or without 1 mM metformin (Met). The OD_{450} values were measured. (O) Growth curves of WT and PRMT6-E164A MDA-MB-468 cells in DMEM medium supplemented with or without 1 mM metformin (Met).

For N-O, data represent means ± SEM; n=3 biological independent experiments, two-tailed paired *t*-tests were used for statistical analysis. *, $P<0.05$; **, $P<0.01$; ***, $P<0.001$; *n.s.*, no significance. For A-F, H, J, K and M, shown are the typical example of three biological independent experiments.

Figure S4. Metformin suppresses cell growth through direct targeting of PRMT6 rather than via non-specific or off-target mechanisms.

(A) Representative flow cytometry plots of Annexin V-FITC/PI staining in WT and PRMT6-E164A MCF7 cells treated with 0.5 mM metformin. The living cell percentage (Annexin V^{-}/PI^{-}), apoptotic cell percentage (Annexin V^{+}/PI^{-} and Annexin V^{+}/PI^{+}) and necrosis cell percentage (Annexin V^{-}/PI^{+}) were calculated from three independent experiments. (B) Analysis the effect of EPZ020411 (10 μM) and MS049 (20 μM) on growth of shCtrl and shPRMT6 MCF7 cells. The EPZ020411 and MS049 groups share the same data of shCtrl and shPRMT6. (C and D) Analysis of the combined use of metformin (Met) and PRMT6 inhibitors (MS049, EPZ020411) on growth of WT and PRMT6-E164A MCF7 cells. 0.5 mM metformin, 10 μM MS049, and 10 μM EPZ020411 were used to treat MCF7 cells.

For A-D, data represent means ± SEM; n=3 biological independent experiments, two-tailed paired *t*-tests were used for statistical analysis. *, $P<0.05$; **, $P<0.01$; ***, $P<0.001$; *n.s.*, no significance.

Figure S5. Metformin represses the transcription of *PRMT6*.

(A) Volcano plots for differentially expressed genes by metformin from RNA-seq experiments. Differential expression levels of aligned sequences were calculated using significant thresholds set at fold change over two and adjusted P value ≤ 0.05. Red color designates significantly up-regulated genes and blue color for significantly down-regulated genes. (B) KEGG analysis of 4,241 genes significantly repressed ($log_2FC \geq 1$, $P \leq 0.05$) by metformin. (C) RT-qPCR analysis of the effect of metformin on the transcription of PRMT family members (PRMT1-PRMT9). (D)

Metformin reduced the levels of PRMT6 and H3R2me2a in MCF7 cells in a dose-dependent manner. Cells were treated with indicated concentration of metformin for 48 h. Bottom panel is the quantification of immunoblots. (E) Effect of metformin on the levels of PRMT6 and H3R2me2a in MDA-MB-436, and MDA-MB-468 cells as determined by immunoblots. Cells were treated with indicated concentration of metformin for 48 h. (F) Effect of metformin on the levels of PRMT6 and H3R2me2a in MDA-MB-231 and HeLa cells as determined by immunoblots. Cells were treated with indicated concentration of metformin for 48 h. (G) Metformin reduced the levels of PRMT6 and H3R2me2a in xenografted MCF7 tumors as determined by immunoblots. Right panel is the quantification of immunoblots.

For C and D, data represent means ± SEM; n=3 biological independent experiments. For G, data represent means ± SEM; n=5 biological independent experiments. Two-tailed *t*-tests were used for statistical analysis. *, $P<0.05$; **, $P<0.01$; ***, $P<0.001$. For E and F, shown are the typical example of two biological independent experiments.

Figure S6. Metformin represses *PRMT6* transcription by inhibiting PRMT6-catalyzed H3R2me2a.

(A) Effect of 0.5 mM metformin (Met) on the levels of PRMT6 and H3R2me2a in control (shCtrl) and *AMPK*-knockdown (shAMPK) MCF7 cells. (B) Inhibition of DNA methylation by 5-Azacytidine (5-AzaC) did not restore metformin-induced reduction of *PRMT6* transcription. MCF7 cells were treated with 0.5 mM metformin with or without 10 μM 5-AzaC for 48 h. (C) In vitro HMT assay showing histone H3R2A mutant reduced the activity of PRMT6 to methylate H3R2 (H3R2me2a). The in vitro HMT assay was performed with 0.4 μg purified PRMT6 and 2

mM WT H3 in the presence of 0-8 mM H3R2A. (D) In vitro HMT assay showing histone H3R2A mutant had no effect on the activity of PRMT4 to methylate H3R17 (H3R17me2a). The in vitro HMT assay was performed with 0.4 μg purified PRMT4 and 2 mM WT H3 in the presence of 0-8 mM H3R2A. (E) Analysis of the levels of histone methyltransferases (PRMT6, PRMT4, EZH2) and histone methylation marks (H3R2me2a, H3R17me2a, H3K27me3, H3K36me3, H3K79me3) in MCF7 cells transfected with plasmids overexpressing WT H3 and H3R2A mutant. (F) Analysis of *PRMT6* transcription in MCF7 cells transfected with plasmids overexpressing WT H3 and H3R2A mutant. (G) Immunoblot analysis of PRMT6 and H3R2me2a in MDA-MB-468 cells overexpressing WT H3 and H3R2A mutant. (H) Immunoblot analysis of the effect of 0.5 mM metformin (Met) on the levels of PRMT6 and H3R2me2a in MDA-MB-468 cells overexpressing WT H3 and H3R2A mutant. (I) Luciferase reporter assay for PRMT6 in *PRMT6*-overexpressing (PRMT6 OE), *PRMT6*-knockdown (shPRMT6) or H3R2A MCF7 cells. (J) Luciferase reporter assay of *PRMT6* promoter fragments spanning regions -2000 to 0 bp, -1500 to 0 bp, -1000 to 0 bp, and -500 to 0 bp in WT MCF7 cells treated with or without 0.5 mM metformin for 48 h. (K) RT-qPCR analysis of *RFX6* and *SPZ1* knockdown efficiency in control (shCtrl), *RFX6*-knockdown (shRFX6) and *SPZ1*-knockdown (shSPZ1) MCF7 cells, respectively. (L) RT-qPCR analysis of *PRMT6* expression in control (shCtrl), *RFX6*-knockdown (shRFX6) and *SPZ1*-knockdown (shSPZ1) MCF7 cells. (M) ChIP-qPCR analysis of SPZ1 occupancy at *PRMT6* promoter in MCF7 cells treated with or without 0.5 mM metformin for 48 h. (N) Analysis of the effect of 1 mM metformin on growth of MCF7 cells stably expressing WT H3 and H3R2A mutant by colony formation assay and CCK-8 cell proliferation assay. (O) Immunoblot analysis of PRMT6 and H3R2me2a in control (Ctrl) and *PRMT6*-overexpressing (PRMT6 OE) MCF7

cells treated with or without 0.5 mM metformin (Met) for 48 h. (P) CCK-8 cell proliferation assay showing overexpression of PRMT6 (PRMT6 OE) attenuated the inhibitory effect of metformin on cell growth. Control (Ctrl) and *PRMT6*-overexpressing (PRMT6 OE) MCF7 cells were grown in MEM medium supplemented with or without 1 mM metformin (Met). (Q-V) Effect of 1 mM metformin (Met) on growth of control (Ctrl), *PRMT1*-overexpression (PRMT1 OE) (M), *PRMT2*-overexpression (PRMT2 OE) (N), *PRMT3*-overexpression (PRMT3 OE) (O), *PRMT5*-overexpression (PRMT5 OE) (P), *PRMT7*-overexpression (PRMT7 OE) (Q) and *PRMT9*-overexpression (PRMT9 OE) (R) MCF7 cells.

For B, E, F, I-N, and P-V, data represent means ± SEM; n=3 biological independent experiments. Two-tailed *t*-tests (B, E, F, I-M) and two-tailed paired *t*-tests (N, P-V) were used for statistical analysis. *, $P<0.05$; **, $P<0.01$; ***, $P<0.001$; *n.s.*, no significance. For A, C, D, G, H and O, shown are the typical example of two biological independent experiments.

Figure S7. Metformin inhibits proliferation of MCF7 independent of SAM/SAH metabolism.

(A) Analysis of the effect of metformin (Met) on SAM/SAH ratio in WT and PRMT6-E164A MCF7 cells. Cells were treated with 0.5 mM metformin for 48 h. (B) Analysis of the effect of metformin (Met) on SAM/SAH ratio in MCF7 cells when treated with 0.5 mM metformin with or without glucose depletion. (C) Immunoblot analysis of PRMT6 and H3R2me2a in MCF7 cells when treated with 0.5 mM metformin with or without glucose depletion. (D) ChIP-qPCR analysis of the effect of metformin on the enrichment of PRMT6 and H3R2me2a at the *PRMT6* promoter under glucose deprivation. (E) Immunoblot analysis of PRMT6 and H3R2me2a in WT and

PRMT6-E164A MCF7 cells when treated with 0.5 mM metformin for 48 h under glucose deprivation. (F) Analysis of the effect of metformin (Met) and SAH on SAM/SAH ratio in MCF7 cells. Cells were treated with 0.5 mM metformin and 50 μM SAH for 48 h. (G) Immunoblot analysis of PRMT6 and H3R2me2a in MCF7 cells when treated with metformin or SAH. (H) Effect of 0.5 mM metformin (Met) and 50 μM SAH on growth of MCF7 cells. (I) Effect of 0.5 mM metformin (Met) on growth of control (shCtrl) and *SHMT2*-knockdown (shSHMT2) MCF7 cells in WT (left) or E164A (right) cells.

For A, B, D, F, H, I, data represent means ± SEM; n=3 biological independent experiments. Two-tailed *t*-tests were used for statistical analysis. *, $P<0.05$; **, $P<0.01$; ***, $P<0.001$. For C, E, G, shown are the typical example of two biological independent experiments.

Figure S8. Metformin induces DNA methylation by inhibiting the activity of PRMT6.

(A) Dot blot analysis of the effect of 0.5 mM metformin (Met) on DNA methylation (5mC) in MCF7 cells for 48 h. Genomic DNA was extracted and DNA methylation was detected by dot blots with anti-5mC antibody. The membrane was stained with SYTOX Green to indicate the loading of genomic DNA. (B) Analysis of the effect of 0.5 mM metformin (+ Met) on DNA methylation in control (shCtrl) and *PRMT6*-knockdown (shPRMT6) MCF7 cells by immunofluorescence. (C) Analysis of the effect of 0.5 mM metformin (+ Met) on DNA methylation in control (shCtrl) and *PRMT6*-knockdown (shPRMT6) MDA-MB-468 cells by immunofluorescence. (D and E) Immunofluorescence and dot blot analysis of the effect of 0.5 mM metformin (Met) on DNA methylation in MCF7 cells overexpressing WT H3 and H3R2A. (F) Immunofluorescence analysis of the effect of 0.5 mM metformin (+ Met) on DNA

methylation in MDA-MB-468 cells overexpressing WT H3 and H3R2A. (G) Subcellular fractionation analysis of the effect of 0.5 mM metformin (+ Met) on the amount of UHRF1 localized in cytoplasm, nuclear matrix and chromatin. (H and I) Analysis of the effect of 0.5 mM metformin (+ Met) on DNA methylation in control (shCtrl), *UHRF1*-knockdown (shUHRF1) and *DNMT1*-knockdown (shDNMT1) MCF7 cells. (J and K) CCK-8 cell proliferation assay showing knockdown of *UHRF1* and *DNMT1* attenuated the inhibitory effect of metformin on cell growth. The shUHRF1 and shDNMT1 groups share the same data of shCtrl and shCtrl+Met. For A, J, K, data represent means ± SEM; n=3 biological independent experiments. Two-tailed *t*-tests (A) and two-tailed paired *t*-tests (J, K) were used for statistical analysis. ***, $P<0.001$. For B-I, shown are the typical example of two biological independent experiments.

Figure S9. Metformin regulates the expression of genes involved in DNA replication.

(A) KEGG analysis of 253 genes exhibiting co-regulation by metformin and PRMT6, along with increased promoter DNA methylation. (B) Bisulfite sequencing analysis of 5mC at the promoter regions of *PKMYT1* and *CDKN2C* in MCF7 cells treated with or without 1 mM metformin (Met) for 48 h. Open circles, unmethylated CpG; filled circles, methylated CpGs. (C) Analysis of the transcription of *PRMT6*, *POLD1*, *POLA2* and *FEN1* from the whole blood transcriptome data for type 2 diabetes (T2D) patients administrated with metformin for three months. Data were retrieved from GSE153792. Two-tailed paired *t*-tests were used for statistical analysis. (D) Analysis of the transcription of *FEN1* and *POLD1* in normal and primary breast tumor tissues from TCGA database. Two-tailed *t*-tests were used for statistical analysis. (E) Analysis of the correlation between the mRNA levels of *PRMT6*, *POLA2*, *FEN1* and *POLD1* in breast cancer

patients of TCGA database. Spearman's rho value was used to evaluate the degree of their correlation. (F) Analysis of the enrichment of 5mC at the promoter regions of *FEN1* and *POLD1* in normal and primary breast tumor tissues from TCGA database. Two-tailed *t*-tests were used for statistical analysis. (G) Effect of the combined treatment of metformin and DNA methylation inhibitor 5-Aza on the transcription of *FEN1*, *POLA2* and *POLD1*. MCF7 cells were treated with 0.5 mM metformin (Met) with or without 10 μM 5-Aza for 48 h. (H) MeDIP analysis of 5mC at the promoter regions of *FEN1*, *POLA2* and *POLD1* in control (Ctrl) and *PRMT6*-overexpressing (PRMT6 OE) MCF7 cells treated with or without 0.5 mM metformin (Met) for 48 h. (I) RT-qPCR analysis of the transcription of *FEN1*, *POLA2* and *POLD1* in control (Ctrl) and *PRMT6*-overexpressing (PRMT6 OE) MCF7 cells treated with or without 0.5 mM metformin (Met) for 48 h. (J-L) RT-qPCR analysis of the transcription of *FEN1*, *POLA2* and *POLD1* in control (shCtrl) and *PRMT6*-knockdown (shPRMT6) MCF7, MDA-MB-436 and MDA-MB-468 cells treated with or without 0.5 mM metformin (Met) for 48 h.

For G-L, data represent means ± SEM; n=3 biological independent experiments. Two-tailed *t*-tests were used for statistical analysis. *, $P<0.05$; **, $P<0.01$; ***, $P<0.001$; *n.s.*, no significance.

Figure S10. Metformin increases DNA methylation to repress gene transcription.

(A and B) RT-qPCR analysis of the transcription of *FEN1*, *POLA2* and *POLD1* in control (shCtrl), *UHRF1*-knockdown (shUHRF1) and *DNMT1*-knockdown (shDNMT1) MCF7 cells treated with or without 0.5 mM metformin (Met) for 48 h. (C) ChIP-qPCR analysis of UHRF1 binding at the promoter regions of *FEN1*, *POLA2* and *POLD1* in MCF7 cells when treated with 0.2 mM metformin for 0-48 h. (D) MeDIP analysis of 5mC at the promoter regions of *FEN1*, *POLA2* and

POLD1 in MCF7 cells when treated with 0.2 mM metformin for 0-48 h. (E) RT-qPCR analysis of the transcription of *FEN1*, *POLA2* and *POLD1* in MCF7 cells when treated with 0.2 mM metformin for 0-48 h. (F) BrdU staining analysis of MCF7 cells when treated with 0.2 mM metformin for 0-48 h. (G) Analysis of the combined use of metformin and hydroxyurea (HU) on growth of WT and PRMT6-E164A MCF7 cells. Cells were treated with 0.2 mM metformin with or without 10 μM HU. (H) Analysis of the combined use of metformin and HU on growth of MDA-MB-436 and MDA-MB-468 cells. Cells were treated with 0.5 mM metformin with or without 10 μM HU. (I) Effect of 0.5 mM metformin (Met) on growth of control (shCtrl), *FEN1*-knockdown (shFEN1), *POLA2*-knockdown (shPOLA2) and *POLD1*-knockdown (shPOLD1) MCF7 cells.

For A-I, data represent means ± SEM; n=3 biological independent experiments. Two-tailed *t*-tests (A-F) and two-tailed paired *t*-tests (G-I) were used for statistical analysis. *, P<0.05; **, P<0.01; ***, P<0.001, *n.s.*, no significance.

Figure S11. Metformin represses tumorigenesis by inhibiting PRMT6.

(A-C) Metformin and PRMT6 inhibitor (MS049) inhibited xenograft growth of MDA-MB-468 cells. The BALB/c nude mice were subcutaneously injected with MDA-MB-468 cells and received PBS (Ctrl), 60 mg/kg metformin (Met), 50 mg/kg MS049, 50 mg/kg 5-AzaC by oral gavage (7 mice/group). The xenograft tumors were measured over time and dissected at the endpoint. Quantification of the average volume of tumors over time was shown in (A). The dissected tumors and quantification of the tumor weight were shown in (B) and (C). (D-F) Metformin inhibited xenograft growth of control (shCtrl) but not *PRMT6*-knockdown (shPRMT6)

MDA-MB-468 cells. The BALB/c nude mice were subcutaneously injected with MDA-MB-468 cells and received PBS (- Met) or 60 mg/kg metformin (+ Met) by oral gavage (10 mice/group). The xenograft tumors were measured over time and dissected at the endpoint. Quantification of the average volume of tumors over time was shown in (D). The dissected tumors and quantification of the tumor weight were shown in (E) and (F). (G) Immunoblot analysis of PRMT6 and H3R2me2a in control (shCtrl) and *PRMT6*-knockdown (shPRMT6) MDA-MB-468-derived tumors administered with PBS (- Met) or metformin (+ Met) in (E). (H) Representative IHC staining images of PRMT6 and H3R2me2a in xenograft tumors administered with PBS (-Met) or metformin (+ Met) in (E). (I) RT-qPCR analysis of the transcription of *FEN1*, *POLA2* and *POLD1* in control (shCtrl) and *PRMT6*-knockdown (shPRMT6) MDA-MB-468-derived tumors administered with PBS (- Met) or metformin (+ Met) in (E).

For A-C, n=7 biological independent experiments; for D-F, n=8 biological independent experiments; for I, n=3 biological independent experiments; data represent means ± SE. Two-tailed *t*-tests (C, F, I) and two-tailed paired *t*-tests (A, D) were used for statistical analysis. *, $P<0.05$; **, $P<0.01$; ***, $P<0.001$, *n.s.*, no significance. For G and H, shown are the typical example of three biological independent experiments.

Figure S12. Correlation analysis of PRMT6 expression in tumor tissues and peripheral blood from breast cancer patients.

The scatter plot showing a positive correlation between PRMT6 expression in tumor tissues and peripheral blood from breast cancer patients. The clinical data were derived from GSE169246.

Figure S1

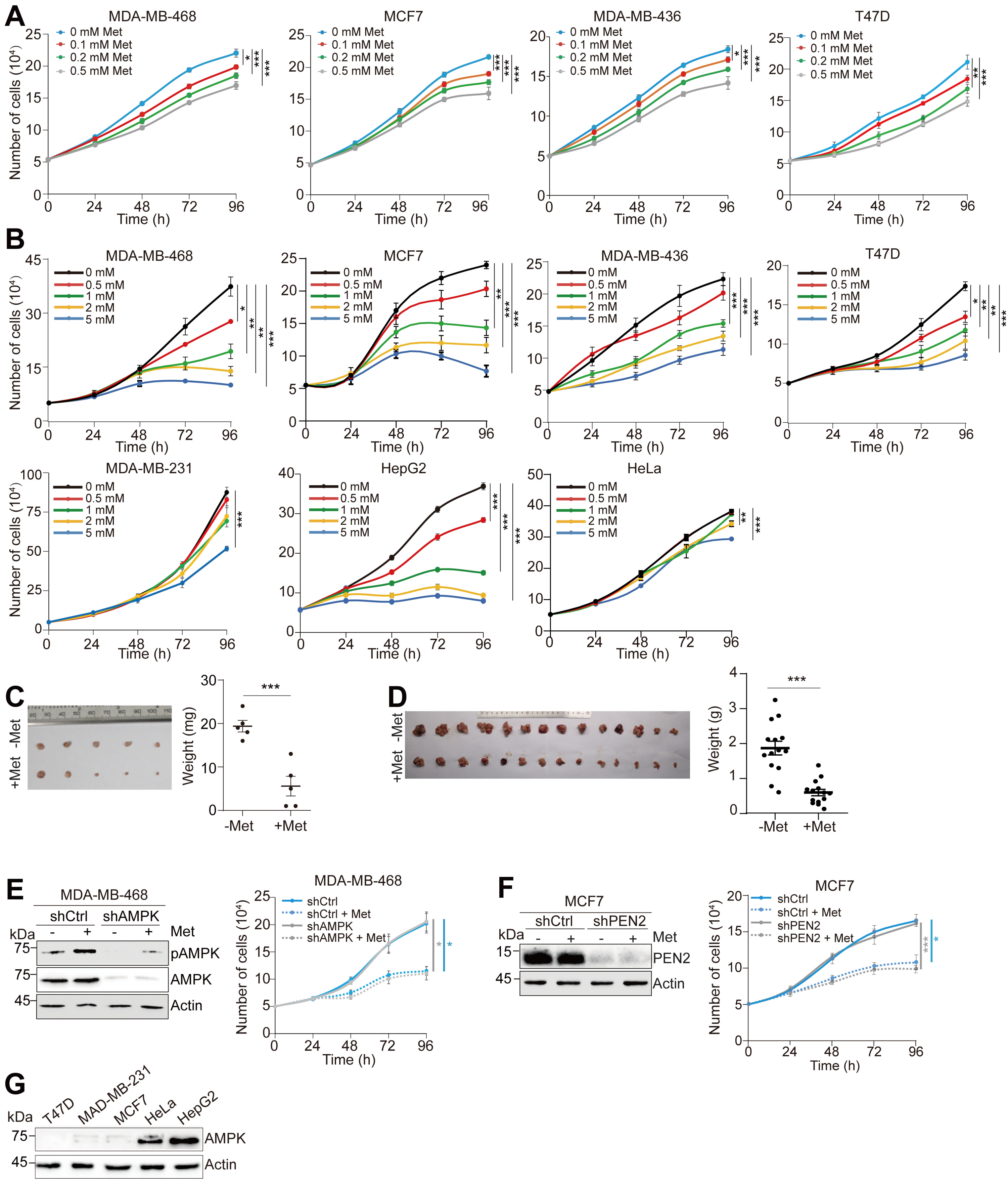
A
MDA-MB-468
MCF7
MDA-MB-436
T47D
0 mM Met
0.1 mM Met
0.2 mM Met
0.5 mM Met
Number of cells (10⁴)
Time (h)
B
MDA-MB-468
MCF7
MDA-MB-436
T47D
MDA-MB-231
HepG2
HeLa
0 mM
0.5 mM
1 mM
2 mM
5 mM
C
-Met
+Met
Weight (mg)
D
Weight (g)
E
MDA-MB-468
shCtrl
shAMPK
kDa
Met
pAMPK
AMPK
Actin
shCtrl + Met
shAMPK + Met
F
MCF7
shPEN2
PEN2
shPEN2 + Met
G
T47D
MAD-MB-231
MCF7
HeLa
HepG2

Figure S2

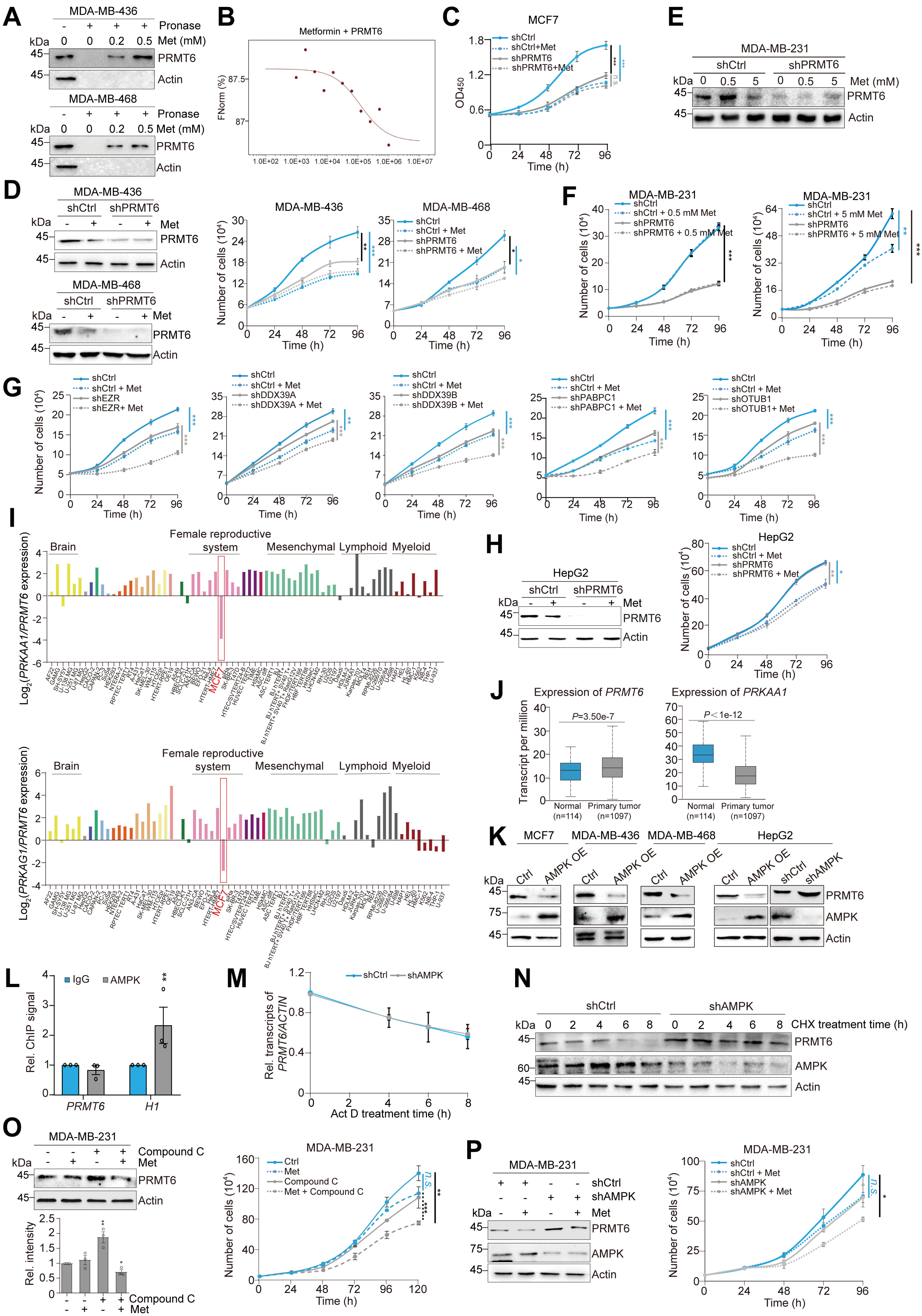

A
MDA-MB-436
Pronase
Met (mM)
PRMT6
Actin
MDA-MB-468
B
Metformin + PRMT6
FNorm (%)
C
MCF7
shCtrl
shCtrl+Met
shPRMT6
shPRMT6+Met
OD450
Time (h)
E
MDA-MB-231
D
Number of cells (10⁴)
F
G
shEZR
shDDX39A
shDDX39B
shPABPC1
shOTUB1
H
HepG2
I
Brain
Female reproductive system
Mesenchymal
Lymphoid
Myeloid
Log2(PRKAA1/PRMT6 expression)
Log2(PRKAG1/PRMT6 expression)
MCF7
J
Expression of PRMT6
Expression of PRKAA1
P=3.50e-7
P<1e-12
Transcript per million
Normal (n=114)
Primary tumor (n=1097)
K
Ctrl
AMPK OE
shAMPK
AMPK
L
IgG
Rel. ChIP signal
PRMT6
H1
M
Rel. transcripts of PRMT6/ACTIN
Act D treatment time (h)
N
CHX treatment time (h)
O
Compound C
Rel. intensity
Ctrl
Met
Met + Compound C
P
shAMPK + Met

Figure S3

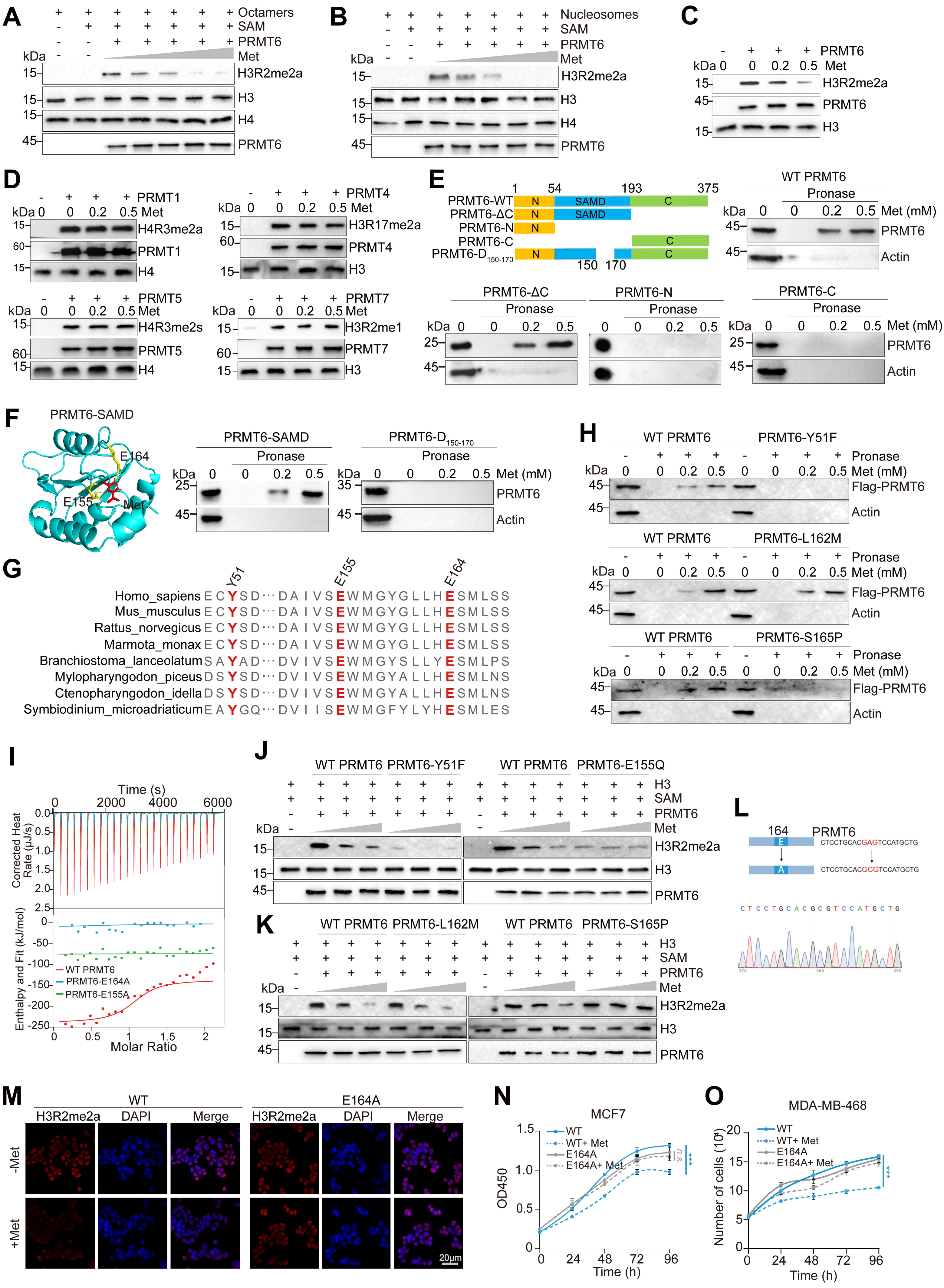

A
Octamers
SAM
PRMT6
Met
kDa
H3R2me2a
H3
H4
PRMT6
B
Nucleosomes
SAM
PRMT6
Met
H3R2me2a
H3
H4
PRMT6
C
PRMT6
Met
0 0.2 0.5
H3R2me2a
PRMT6
H3
D
PRMT1
H4R3me2a
PRMT1
H4
PRMT4
H3R17me2a
PRMT4
H3
PRMT5
H4R3me2s
PRMT5
H4
PRMT7
H3R2me1
PRMT7
H3
E
PRMT6-WT
PRMT6-ΔC
PRMT6-N
PRMT6-C
PRMT6-D150-170
N
SAMD
C
1 54 193 375
150 170
WT PRMT6
Pronase
Met (mM)
PRMT6
Actin
PRMT6-ΔC
PRMT6-N
PRMT6-C
F
PRMT6-SAMD
E164
E155
Met
PRMT6-D150-170
Pronase
Met (mM)
PRMT6
Actin
G
Y51
E155
E164
Homo_sapiens E C Y S D ··· D A I V S E W M G Y G L L H E S M L S S
Mus_musculus E C Y S D ··· D A I V S E W M G Y G L L H E S M L S S
Rattus_norvegicus E C Y S D ··· D A I V S E W M G Y G L L H E S M L S S
Marmota_monax E C Y S D ··· D A I V S E W M G Y G L L H E S M L S S
Branchiostoma_lanceolatum S A Y A D ··· D V I V S E W M G Y S L L Y E S M L P S
Mylopharyngodon_piceus D S Y S D ··· D V I V S E W M G Y A L L H E S M L N S
Ctenopharyngodon_idella D S Y S D ··· D V I V S E W M G Y A L L H E S M L N S
Symbiodinium_microadriaticum E A Y G Q ··· D V I I S E W M G F Y L Y H E S M L E S
H
WT PRMT6
PRMT6-Y51F
PRMT6-L162M
PRMT6-S165P
Pronase
Met (mM)
Flag-PRMT6
Actin
I
Time (s)
Corrected Heat Rate (μJ/s)
Enthalpy and Fit (kJ/mol)
WT PRMT6
PRMT6-E164A
PRMT6-E155A
Molar Ratio
J
WT PRMT6
PRMT6-Y51F
PRMT6-E155Q
H3
SAM
PRMT6
Met
H3R2me2a
H3
PRMT6
K
WT PRMT6
PRMT6-L162M
PRMT6-S165P
H3R2me2a
H3
PRMT6
L
164
PRMT6
E
A
CTCCTGCACGAGTCCATGCTG
CTCCTGCACGCGTCCATGCTG
M
WT
E164A
H3R2me2a
DAPI
Merge
-Met
+Met
20μm
N
MCF7
OD450
Time (h)
WT
WT+ Met
E164A
E164A+ Met
n.s.
***
O
MDA-MB-468
Number of cells (10⁴)
Time (h)
WT
WT+ Met
E164A
E164A+ Met
***

Figure S4

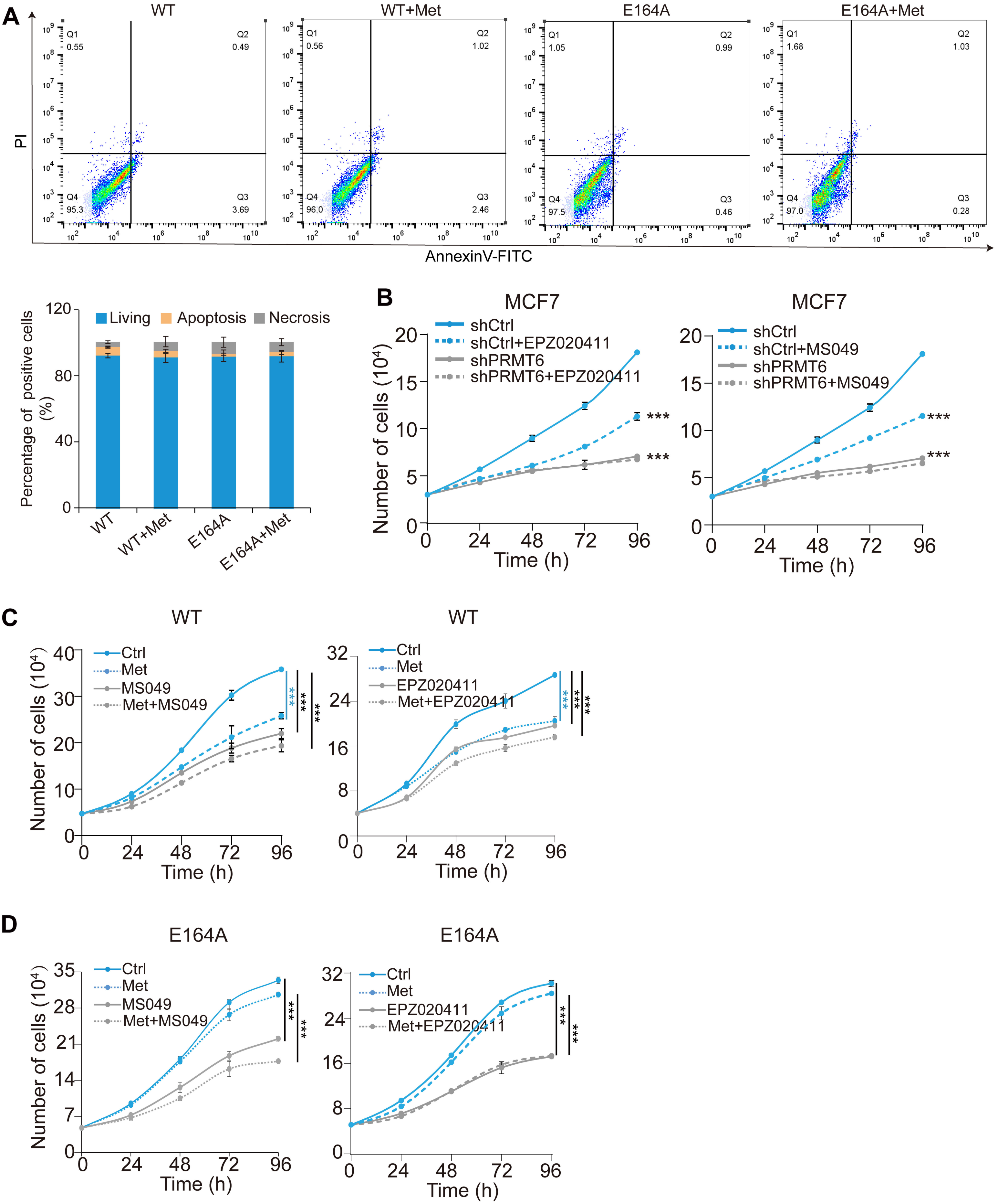
A
WT
WT+Met
E164A
E164A+Met
PI
AnnexinV-FITC
Q1 0.55
Q2 0.49
Q4 95.3
Q3 3.69
Q1 0.56
Q2 1.02
Q4 96.0
Q3 2.46
Q1 1.05
Q2 0.99
Q4 97.5
Q3 0.46
Q1 1.68
Q2 1.03
Q4 97.0
Q3 0.28
Living
Apoptosis
Necrosis
Percentage of positive cells (%)
B
MCF7
shCtrl
shCtrl+EPZ020411
shPRMT6
shPRMT6+EPZ020411
shCtrl+MS049
shPRMT6+MS049
Number of cells (10^4)
Time (h)
C
WT
Ctrl
Met
MS049
Met+MS049
EPZ020411
Met+EPZ020411
D
E164A

Figure S5

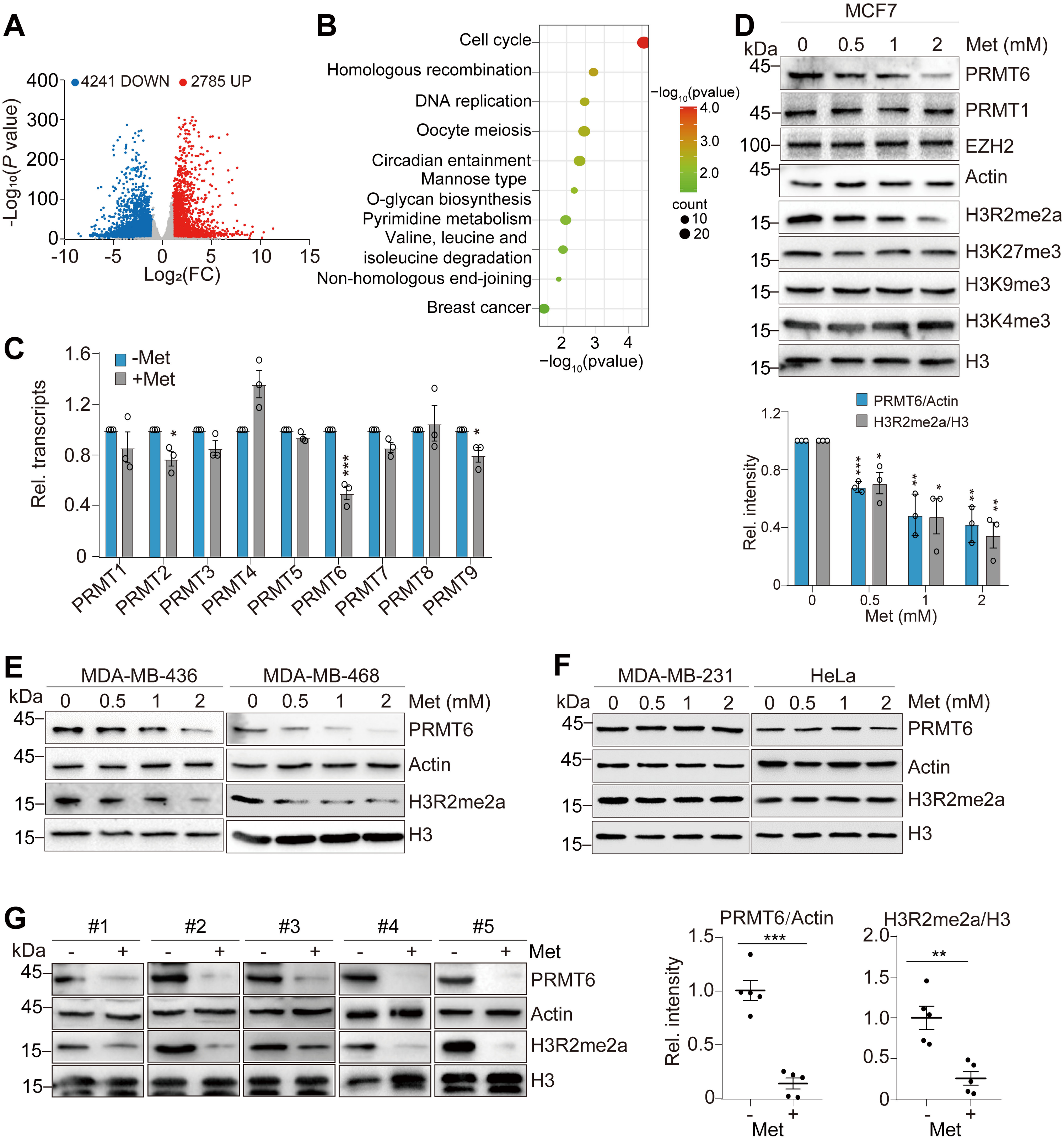

A
4241 DOWN
2785 UP
-Log10(P value)
Log2(FC)
B
Cell cycle
Homologous recombination
DNA replication
Oocyte meiosis
Circadian entainment
Mannose type O-glycan biosynthesis
Pyrimidine metabolism
Valine, leucine and isoleucine degradation
Non-homologous end-joining
Breast cancer
−log10(pvalue)
count
C
-Met
+Met
Rel. transcripts
PRMT1
PRMT2
PRMT3
PRMT4
PRMT5
PRMT6
PRMT7
PRMT8
PRMT9
D
MCF7
kDa
Met (mM)
PRMT6
PRMT1
EZH2
Actin
H3R2me2a
H3K27me3
H3K9me3
H3K4me3
H3
PRMT6/Actin
H3R2me2a/H3
Rel. intensity
Met (mM)
E
MDA-MB-436
MDA-MB-468
Met (mM)
PRMT6
Actin
H3R2me2a
H3
F
MDA-MB-231
HeLa
Met (mM)
PRMT6
Actin
H3R2me2a
H3
G
#1
#2
#3
#4
#5
Met
PRMT6
Actin
H3R2me2a
H3
PRMT6/Actin
H3R2me2a/H3
Rel. intensity
Met

Figure S6

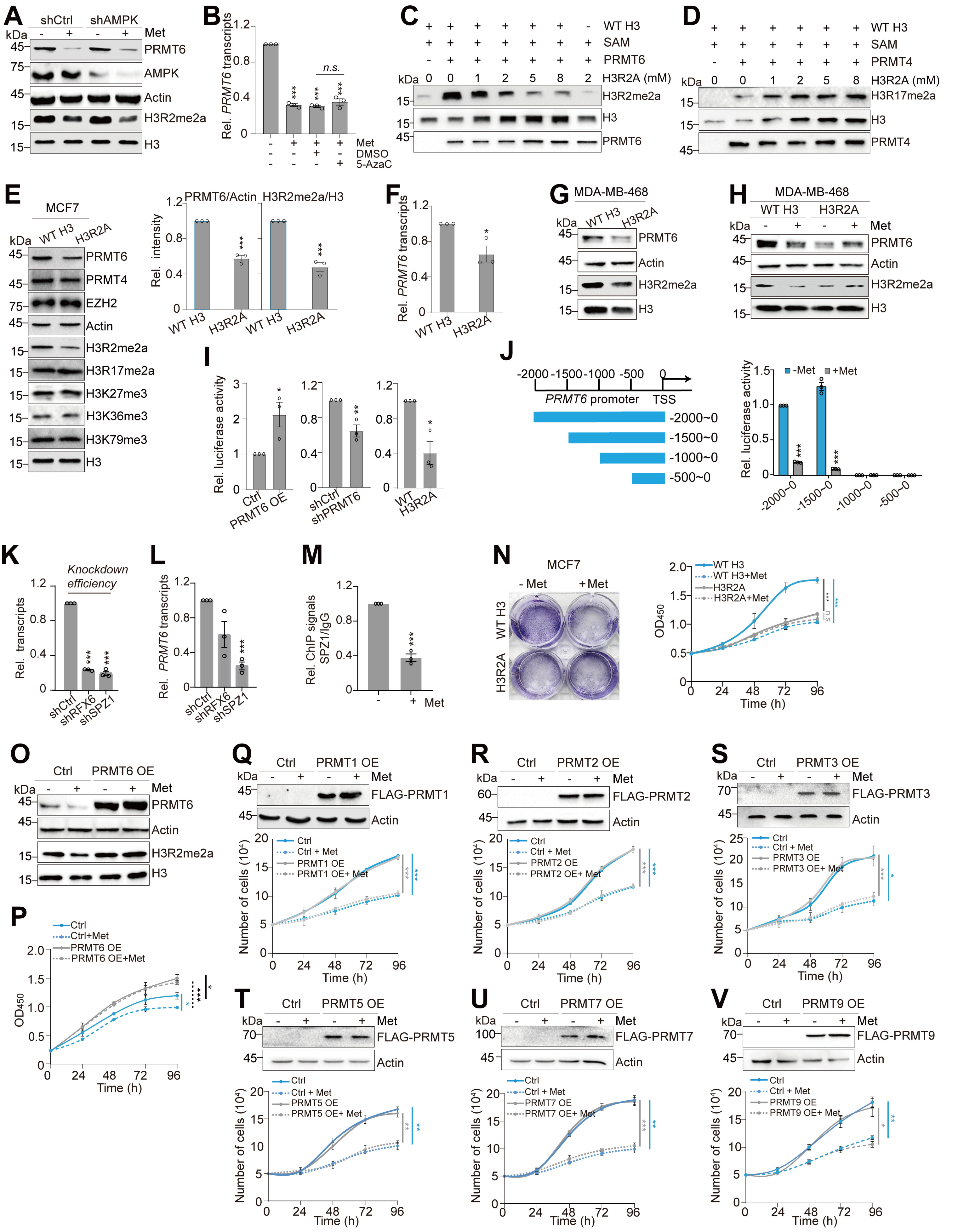
A
shCtrl
shAMPK
Met
kDa
PRMT6
AMPK
Actin
H3R2me2a
H3
B
Rel. PRMT6 transcripts
n.s.
Met
DMSO
5-AzaC
C
WT H3
SAM
PRMT6
H3R2A (mM)
H3R2me2a
H3
PRMT6
D
WT H3
SAM
PRMT4
H3R2A (mM)
H3R17me2a
H3
PRMT4
E
MCF7
WT H3
H3R2A
PRMT6
PRMT4
EZH2
Actin
H3R2me2a
H3R17me2a
H3K27me3
H3K36me3
H3K79me3
H3
PRMT6/Actin
H3R2me2a/H3
Rel. intensity
F
Rel. PRMT6 transcripts
G
MDA-MB-468
H
MDA-MB-468
I
Rel. luciferase activity
Ctrl
PRMT6 OE
shCtrl
shPRMT6
WT
H3R2A
J
PRMT6 promoter
TSS
-2000~0
-1500~0
-1000~0
-500~0
-Met
+Met
Rel. luciferase activity
K
Knockdown efficiency
Rel. transcripts
shCtrl
shRFX6
shSPZ1
L
Rel. PRMT6 transcripts
M
Rel. ChIP signals SPZ1/IgG
Met
N
MCF7
- Met
+Met
WT H3
H3R2A
WT H3+Met
H3R2A+Met
OD450
Time (h)
O
Ctrl
PRMT6 OE
P
Ctrl+Met
PRMT6 OE+Met
Q
PRMT1 OE
FLAG-PRMT1
Number of cells (10^4)
R
PRMT2 OE
FLAG-PRMT2
S
PRMT3 OE
FLAG-PRMT3
T
PRMT5 OE
FLAG-PRMT5
U
PRMT7 OE
FLAG-PRMT7
V
PRMT9 OE
FLAG-PRMT9

Figure S7

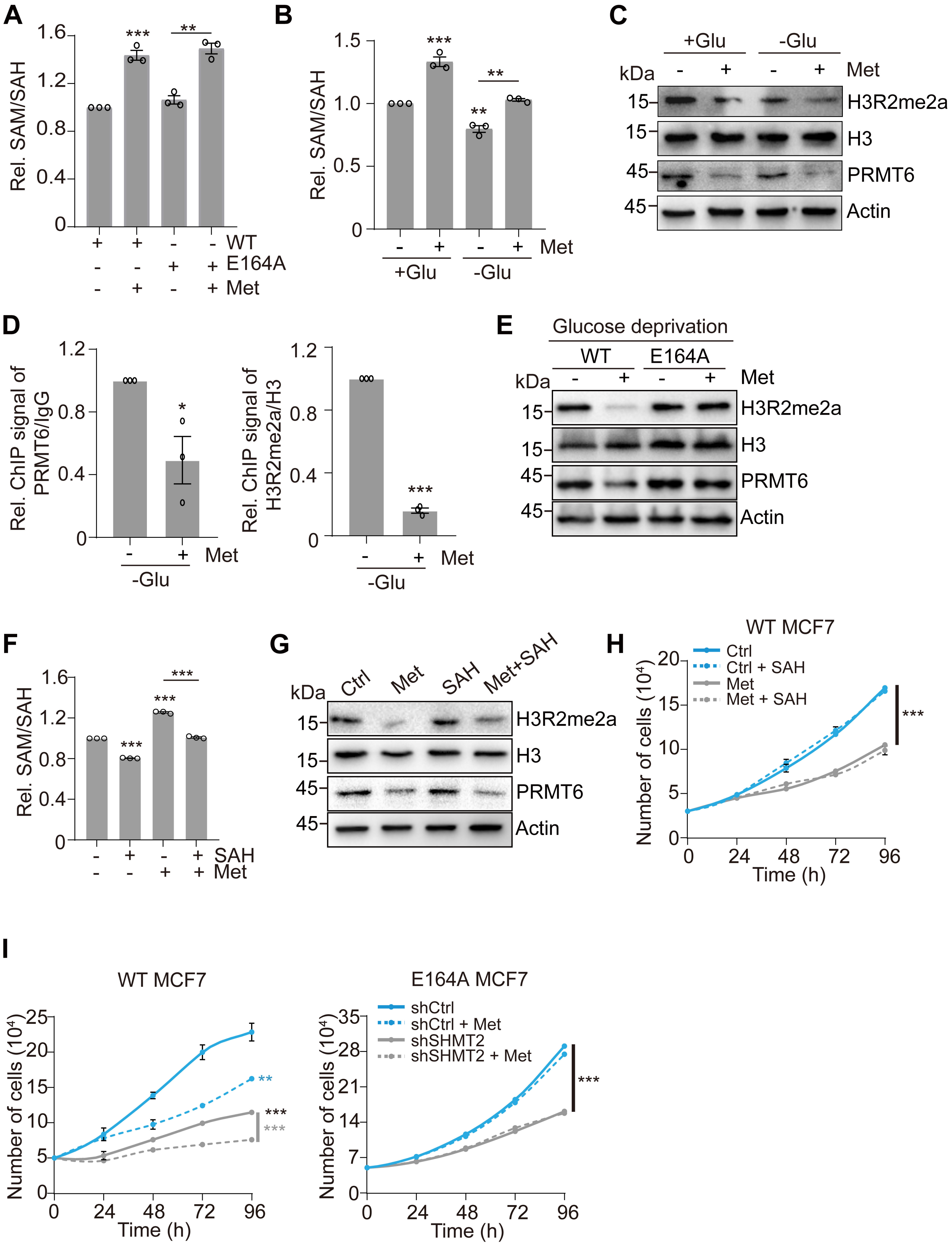
A
Rel. SAM/SAH
WT
E164A
Met
B
Rel. SAM/SAH
Met
+Glu
-Glu
C
+Glu
-Glu
kDa
Met
H3R2me2a
H3
PRMT6
Actin
D
Rel. ChIP signal of PRMT6/IgG
Rel. ChIP signal of H3R2me2a/H3
Met
-Glu
E
Glucose deprivation
WT
E164A
kDa
Met
H3R2me2a
H3
PRMT6
Actin
F
Rel. SAM/SAH
SAH
Met
G
kDa
Ctrl
Met
SAH
Met+SAH
H3R2me2a
H3
PRMT6
Actin
H
WT MCF7
Ctrl
Ctrl + SAH
Met
Met + SAH
Number of cells (10^4)
Time (h)
I
WT MCF7
Number of cells (10^4)
Time (h)
E164A MCF7
shCtrl
shCtrl + Met
shSHMT2
shSHMT2 + Met
Number of cells (10^4)
Time (h)

Figure S8

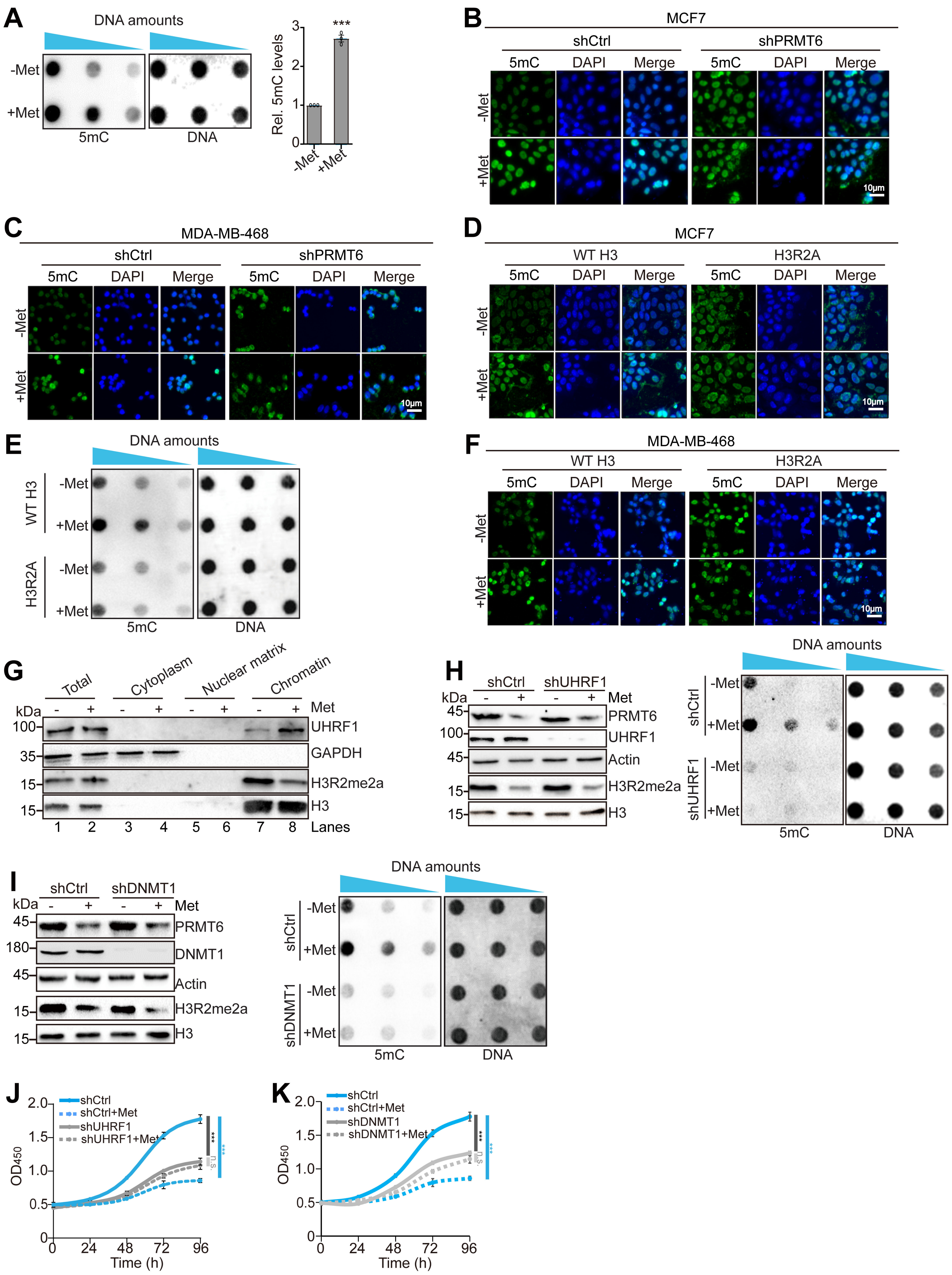
A
DNA amounts
-Met
+Met
5mC
DNA
Rel. 5mC levels
***
B
MCF7
shCtrl
shPRMT6
5mC
DAPI
Merge
10μm
C
MDA-MB-468
D
WT H3
H3R2A
E
F
G
Total
Cytoplasm
Nuclear matrix
Chromatin
kDa
Met
UHRF1
GAPDH
H3R2me2a
H3
Lanes
H
shUHRF1
PRMT6
Actin
I
shDNMT1
DNMT1
J
shCtrl+Met
shUHRF1
shUHRF1+Met
OD450
Time (h)
n.s.
K
shDNMT1+Met

Figure S9

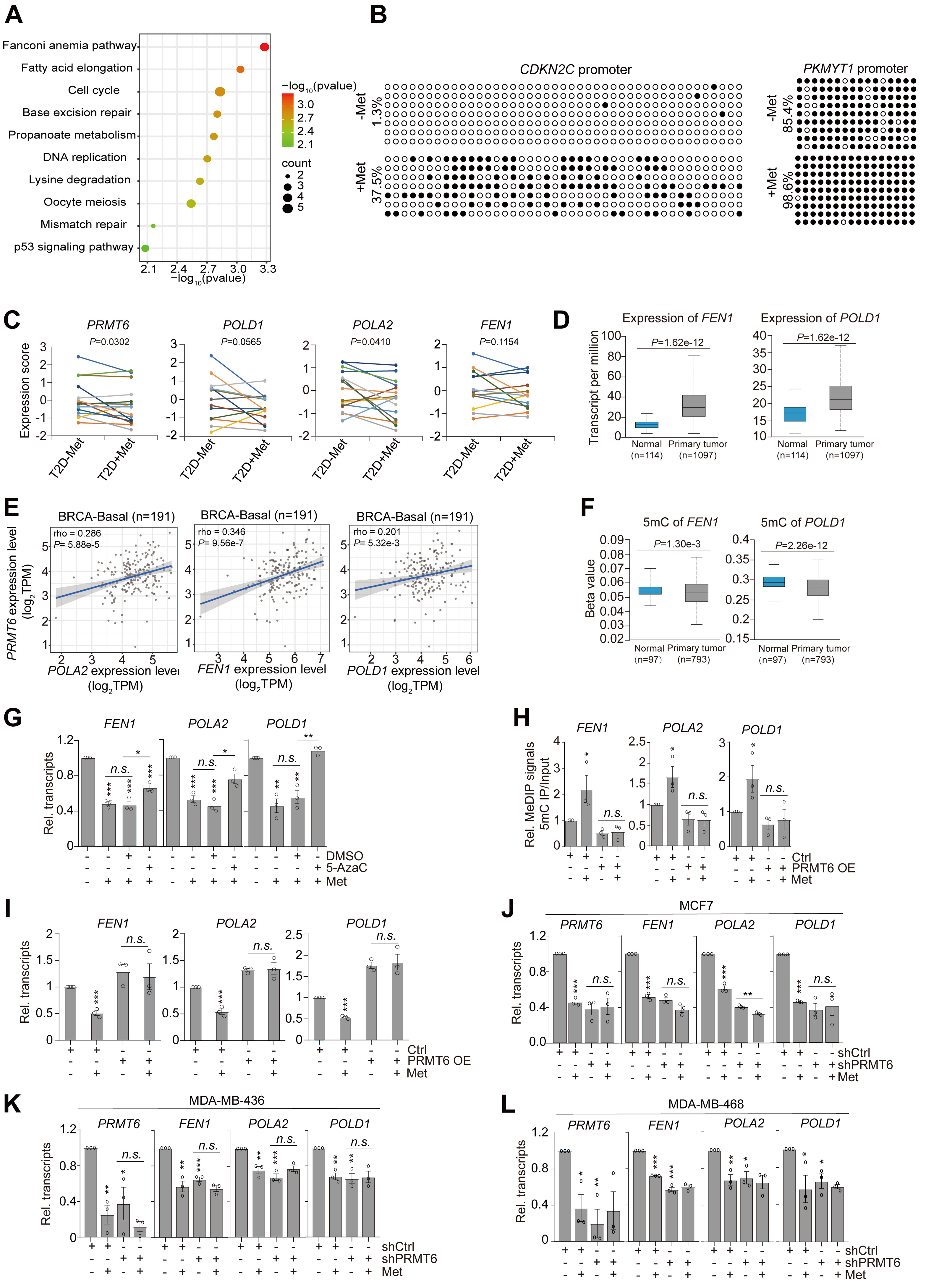

A
Fanconi anemia pathway
Fatty acid elongation
Cell cycle
Base excision repair
Propanoate metabolism
DNA replication
Lysine degradation
Oocyte meiosis
Mismatch repair
p53 signaling pathway
count
B
CDKN2C promoter
PKMYT1 promoter
-Met 1.3%
+Met 37.5%
-Met 85.4%
+Met 98.6%
C
PRMT6 P=0.0302
POLD1 P=0.0565
POLA2 P=0.0410
FEN1 P=0.1154
Expression score
T2D-Met
T2D+Met
D
Expression of FEN1
Expression of POLD1
P=1.62e-12
Transcript per million
Normal (n=114)
Primary tumor (n=1097)
E
BRCA-Basal (n=191)
rho = 0.286 P= 5.88e-5
rho = 0.346 P= 9.56e-7
rho = 0.201 P= 5.32e-3
PRMT6 expression level (log2TPM)
POLA2 expression level (log2TPM)
FEN1 expression level (log2TPM)
POLD1 expression level (log2TPM)
F
5mC of FEN1
5mC of POLD1
P=1.30e-3
P=2.26e-12
Beta value
Normal (n=97)
Primary tumor (n=793)
G
FEN1
POLA2
POLD1
Rel. transcripts
DMSO
5-AzaC
Met
H
Rel. MeDIP signals 5mC IP/Input
Ctrl
PRMT6 OE
Met
I
J
MCF7
PRMT6
shCtrl
shPRMT6
K
MDA-MB-436
L
MDA-MB-468

Figure S10

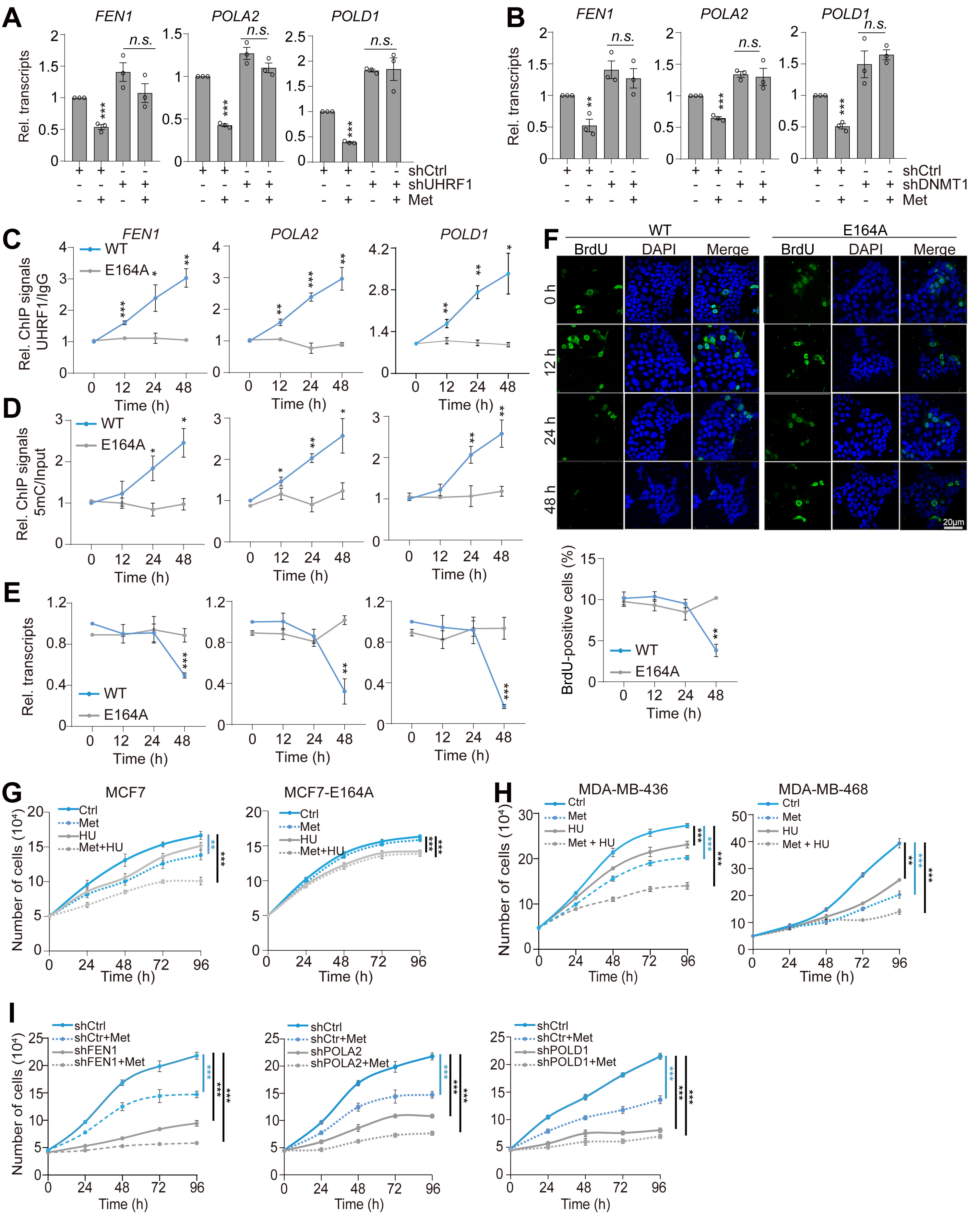
A
FEN1
POLA2
POLD1
Rel. transcripts
n.s.
shCtrl
shUHRF1
Met
B
shDNMT1
C
WT
E164A
Rel. ChIP signals UHRF1/IgG
Time (h)
D
Rel. ChIP signals 5mC/Input
E
F
BrdU
DAPI
Merge
0 h
12 h
24 h
48 h
20μm
BrdU-positive cells (%)
G
MCF7
MCF7-E164A
Ctrl
Met
HU
Met+HU
Number of cells (10⁴)
H
MDA-MB-436
MDA-MB-468
Met + HU
I
shCtrl
shCtr+Met
shFEN1
shFEN1+Met
shPOLA2
shPOLA2+Met
shPOLD1
shPOLD1+Met

Figure S11

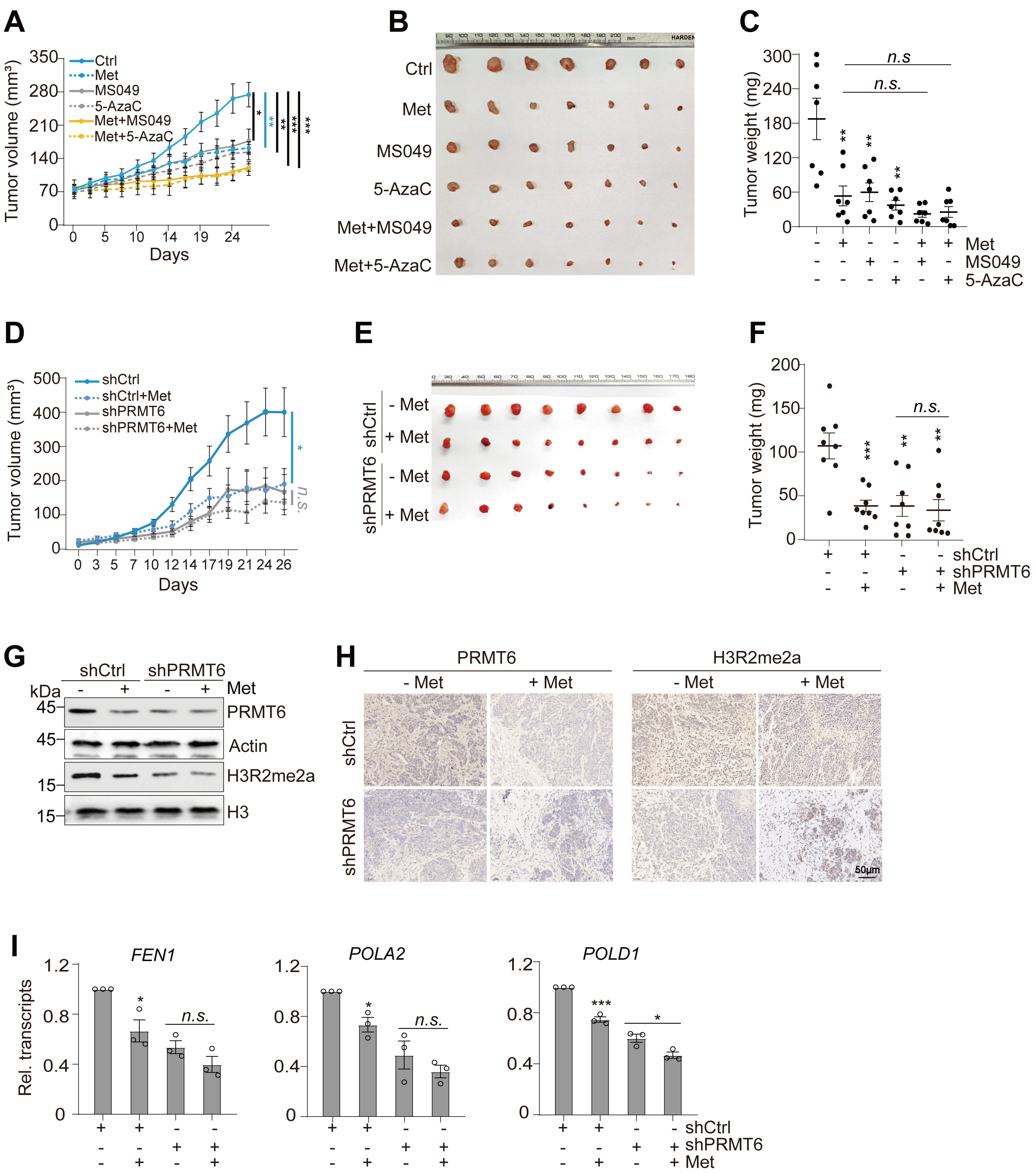
A
Ctrl
Met
MS049
5-AzaC
Met+MS049
Met+5-AzaC
Tumor volume (mm³)
Days
B
C
Tumor weight (mg)
n.s
n.s.
Met
MS049
5-AzaC
D
shCtrl
shCtrl+Met
shPRMT6
shPRMT6+Met
Tumor volume (mm³)
Days
n.s.
E
shCtrl
shPRMT6
- Met
+ Met
F
Tumor weight (mg)
n.s.
shCtrl
shPRMT6
Met
G
shCtrl
shPRMT6
kDa
Met
PRMT6
Actin
H3R2me2a
H3
H
PRMT6
H3R2me2a
- Met
+ Met
shCtrl
shPRMT6
50μm
I
FEN1
POLA2
POLD1
Rel. transcripts
n.s.
shCtrl
shPRMT6
Met

Figure S12

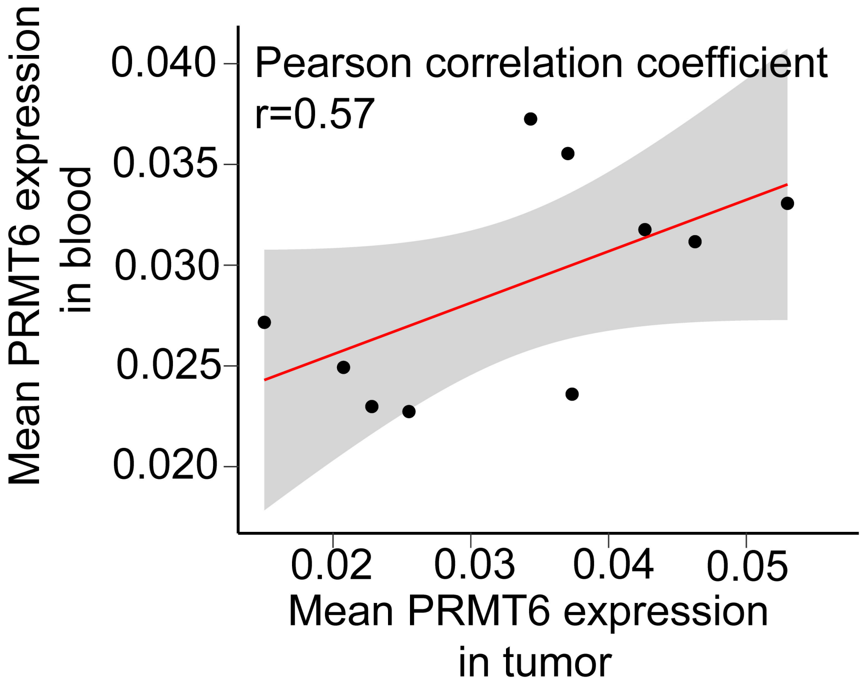
Pearson correlation coefficient
r=0.57
0.040
0.035
0.030
0.025
0.020
Mean PRMT6 expression
in blood
0.02
0.03
0.04
0.05
Mean PRMT6 expression
in tumor

Supplemental Table 1. List of siRNA and shRNA used in this study

Gene name	Sequences
PRMT6#1	5'- CCGGCAACGGATACAGCGTGCTTATCTCGA GATAAGCACGCTGTATCCGTTG TTTTTG -3'
	5'- AATTCAAAAACAACGGATACAGCGTGCTTA TCTCGAGATAAGCACGCTGTATCCGTTG -3'
PRMT6#2	5'- CCGGCACCGGCATTCTGAGCATCTTCTCGAG AAGATGCTCAGAATGCCGGTGTTTTTG -3'
	5'- AATTCAAAAACACCGGCATTCTGAGCATCTT CTCGAGAAGATGCTCAGAATGCCGGTG -3'
AMPKα1/PRKAA1	5'- CCGGGGATTATTGTCACAGGCATTTCAAGAG AATGCCTGTGACAATAATCCTTTTTT -3'
	5'- AATTAAAAAAGGATTATTGTCACAGGCATTCT CTTGAAATGCCTGTGACAATAATCC -3'
AMPKα2/PRKAA2	5'- CCGGGGCTCTTTCAGCAGATTCTTTCAAGAGA AGAATCTGCTGAAAGAGCCTTTTTT -3'
	5'- AATTAAAAAAGGCTCTTTCAGCAGATTCTTCTC TTGAAAGAATCTGCTGAAAGAGCC -3'
UHRF1	5'- CCGGAGATATAACGTTAGGGTTTCTCGAGAAAC CCTAACGTTATATCT TTTTTG -3'
	5'- AATTCAAAAAAGATATAACGTTAGGGTTTCTCG AGAAACCCTAACGTTATATCT -3'
DNMT1	5'- CCGGGCAGGCGGCTCAAAGATTTGTTCAAGAGA CAAATCTTTGAGCCGCCTGCTTTTTC -3'
	5'- AATTGAAAAAGCAGGCGGCTCAAAGATTTGTCTCT TGAACAAATCTTTGAGCCGCCTGC -3'
DDX39A	5'- CCGGGCTGGAGTTTAACCAGGTGATCTCGAGATCA CCTGGTTAAACTCCAGCTTTTTG -3'
	5'- AATTCAAAAAGCTGGAGTTTAACCAGGTGAT CTCGAG ATCACCTGGTTAAACTCCAGC -3'
DDX39B	5'- CCGGTAGACATCTCCTCCTACATCTCGAGATG TAGGAGGAGATGTCTATTTTTG -3'
	5'- AATTCAAAAATAGACATCTCCTCCTACATCTCGAG ATGTAGGAGGAGATGTCTA -3'
PABPC1	5'-CCGGCTAGCCAAATTGCTCAACTACTCGAGTAGT TGAGCAATTTGGCTAGTTTTTG-3'
	5'-AATTCAAAAACTAGCCAAATTGCTCAACTACTCGAG TAGTTGAGCAATTTGGCTAG-3'
EZR	5'- CCGGTCTCTCTCCACGGTTTCTCTCCTCGAGGAGAG AAACCGTGGAGAGAGATTTTTG -3'

	5’- AATT CAAAAA TCTCTCTCCACGGTTTCTCTCCTCG AGGAGAGAAACCGTGGAGAGAGA -3’
OTUB1	5’- CCGGGACCAGGCCTGACGGCAACCTCGAGGTTG CCGTCAGGCCTGGTCTTTTTG-3’
	5’-AATTCAAAAA GACCAGGCCTGACGGCAACCTCGAG GTTGCCGTCAGGCCTGGTC-3’
Spz1	5’-CCGGCCATTGCCTTATTCGAAATCTCGAGATTTCGA ATAAGGCAATGGTTTTTG-3’
	5’-AATTCAAAAACCATTGCCTTATTCGAAATCTCGAGA TTTCGAATAAGGCAATGG-3’
Rfx6	5’-CCGGGCACTTAAACAATGGTAACCTCGAGGTTACCAT TGTTTAAGTGCTTTTTG-3’
	5’-AATTCAAAAAGCACTTAAACAATGGTAACCTCGAG GTTACCATTGTTTAAGTGC-3’
PEN2 (siRNA)	5’- CAATAAAGGACCCTAACTT -3’

Supplemental Table 2. List of oligonucleotides used in this study

Gene name	Sequences
ChIP-qPCR/MeDIP-qPCR	
PRMT6 promoter	CAGAATGTGGGCGTCGTG
	CCTACTTCCTGCGGGGTC
FEN1 promoter	GAACTCCCCTCAACGCTC
	ACAAGTCCCTCAATGCCAC
POLA2 promoter	TTTAGATAGTCGAAGGCACGC
	GATAAAACTCAGTGGCGCATG
POLD1 promoter	GAGGGTGGGAGGAGAGAG
	CATGACCTGTCTGCCCG
H1 promoter	TGTCGGAGACTGCTCCACTT
	GTCCGGATGCTTTGCGTTTC
qRT-PCR	
ACTIN	GCCGACAGGATGCAGAAGGAGATCA
	AAGCATTTGCGGTGGACGATGGA
PRMT6	AGACACGGACGTTTCAGGAG
	CCACTTTGTAGCGCAGCAG
FEN1	ATCCCTTATCTTGATGCACCC
	AGTCAGGTGTCGCATTAGC
POLA2	GCATGAACAGGTGGAGAATTG
	ATGACGGGACAAAGACAAGG
POLD1	GAGATGGAGGCAGAACACAG
	GAGGATCTATGGCTGATGGTG
PEN2	GTGGTTGTTCGTGATCCTTGC
	GACACTCGCTCCAGGTTCAT
PRMT1	TGACTCCTACGCACACTTTG
	ATGGAGTTGCGGTAAGTGAG
PRMT2	ATCCTGAGACAAACCACTGC
	GTACTCTTCATCCTGCCACG
PRMT3	TGAAACATATGGAAGCCAGGG
	GTAGATGACGAGCAGGTTCTG
PRMT4	CAAAACCACACCGACTTCAAG
	CGTAGATTTTCCGTGCTCCAG
PRMT5	GAGATGCCTTATGTGGTACGG
	GCTTAATAGGGAAGAGGATGGG
PRMT7	GTGAAGGACAGAGGACAGAAG
	CTCGATGGCATAGCAGAAGTC
PRMT8	AAGTGGCTGAAACCTGGAG
	TGTCTTCAATCGCTACCACG
PRMT9	GTGTCTTGGGTTTGGAATGTG
	AGCATCTGGTTTACTGGTCTG
Spz1	ACAGGAAGCAGACACCTGGA
	AAAGCTGCCATCAACTGCAA
Rfx6	GAACAAGAGGCCATTCAAAG
	TCCAGTTTTTGAGCTAAGCG
mACTIN	GTGACGTTGACATCCGTAAAGA
	GCCGGACTCATCGTACTCC
mPRMT6	GGGAGACCATGAGGAAAAGAC
	GAGCGGTGAGGTAAAAGAGAC

mPOLA2	CACTTTATCCACCCCACGAG
	ACACAGACACAGCCAAAGATG
mPOLD1	GTATCCAGTCACTTCCCATCAC
	CCCTTGCAGTCCATTTTGTC
mFEN1	CAAGGAGCCAGAACCCAAG
	GCCGAGGGTACAAAACAGATAG

Supplemental Table 3. List of antibodies used in this study

Antibodies	Source	Catalog number
Anti-H3	Abcam	CAT#ab1791
Anti-H4	Abcam	CAT#ab10158
Anti-UHRF1	Millipore	CAT#MABE308
Anti-5mC	Millipore	CAT#MABE146
Anti-pAMPK	Cell Signaling Technology	CAT#2535
Anti-AMPKα	Cell Signaling Technology	CAT#2532
Anti-PRMT6	Proteintech	CAT#15395-1-AP
Anti-GAPDH	Proteintech	CAT#10494-1-AP
Anti-Beta-actin	Proteintech	CAT#20536-1-AP
Peroxidase-conjugated Affinipure Goat anti-Mouse IgG(H+L)	Proteintech	CAT#SA00001-1
Peroxidase-conjugated Affinipure Goat anti-Rabbit IgG(H+L)	Proteintech	CAT#SA000012
Anti-Spz1	Proteintech	CAT#18931-1-AP
Anti-H3R2me2a	Abclonal	CAT#A3155
Anti-PEN2	Abclonal	CAT#A8678
Anti-H3K4me3	Abclonal	CAT#A2357
Anti-H3K9me3	Abclonal	CAT#A2360
Anti-H3K27me3	Abclonal	CAT#A2363
Alexa Fluor 594-conjugated Goat Anti-Rabbit IgG (H+L)	Abclonal	CAT# AS039
Alexa Fluor 488-conjugated Goat Anti-Mouse IgG (H+L)	Abclonal	CAT# AS037
Anti-H4R3me2a	Abclonal	CAT#A2376
Anti-H3R2me1	Abclonal	CAT#A3154
Anti-H4R3me2s	ACTIVE MOTIF	CAT#61188
Anti-FLAG M2	Sigma-Aldrich	CAT#F1804-1MG
Anti-PRMT4	ZenBioscience	CAT#200135
Anti-DNMT1	Wanleibo	CAT#WL02772
